# Chronic Pain and Mortality: A Systematic Review

**DOI:** 10.1371/journal.pone.0099048

**Published:** 2014-06-05

**Authors:** Diane Smith, Ross Wilkie, Olalekan Uthman, Joanne L. Jordan, John McBeth

**Affiliations:** 1 Arthritis Research UK Primary Care Centre, Research Institute for Primary Care and Health Sciences, Keele University, Staffordshire, United Kingdom; 2 Warwick Centre for Applied Health Research and Delivery (WCAHRD), Division of Health Sciences, Warwick Medical School, University of Warwick, Coventry, United Kingdom; NIH - National Institute of Environmental Health Sciences, United States of America

## Abstract

**Background:**

Chronic pain is common, often widespread and has a substantial impact on health and quality of life. The relationship between chronic pain and mortality is unclear. This systematic review aimed to identify and evaluate evidence for a relationship between chronic pain and mortality.

**Methods:**

A search of ten electronic databases including EMBASE and MEDLINE was conducted in March 2012, and updated until March 2014. Observational studies investigating the association between chronic or widespread pain (including fibromyalgia) and mortality were included. Risk of bias was assessed and a meta-analysis was undertaken to quantify heterogeneity and pool results. A narrative review was undertaken to explore similarities and differences between the included studies.

**Results:**

Ten studies were included in the review. Three reported significant associations between chronic or widespread pain and mortality in unadjusted results. In adjusted analyses, four studies reported a significant association. The remaining studies reported no statistically significant association. A meta-analysis showed statistically significant heterogeneity of results from studies using comparable outcome measures (n = 7)(I^2^ = 78.8%) and a modest but non-significant pooled estimate (MRR1.14,95%CI 0.95–1.37) for the relationship between chronic pain and all-cause mortality. This association was stronger when analysis was restricted to studies of widespread pain (n = 5,I^2^ = 82.3%) MRR1.22(95%CI 0.93–1.60). The same pattern was observed with deaths from cancer and cardiovascular diseases. Heterogeneity is likely to be due to differences in study populations, follow-up time, pain phenotype, methods of analysis and use of confounding factors.

**Conclusion:**

This review showed a mildly increased risk of death in people with chronic pain, particularly from cancer. However, the small number of studies and methodological differences prevented clear conclusions from being drawn. Consistently applied definitions of chronic pain and further investigation of the role of health, lifestyle, social and psychological factors in future studies will improve understanding of the relationship between chronic pain and mortality.

## Introduction

Musculoskeletal pain is one of the most common complaints in adults [1]. It has a major impact on physical and mental health, daily activities and is a frequent reason for health care consultation [2]–[4]. Musculoskeletal pain may be associated with increased mortality although the relationship is unclear. Studies of single site pain (e.g. back pain [5], [6]), hip and shoulder [7], [8]) and simple counts of the number of pain sites [9], [10] have produced conflicting results. A lack of consistency in case definitions of pain makes it difficult to compare studies and may explain some of the variation in findings.

Chronic pain may be a more useful starting point to examine the relationship between pain and mortality. There is potential for greater uniformity in case definition with use of recognised criteria [11]. Chronic pain, that is pain that lasts for three months or longer [11], is experienced by up to 30% of adults [12] and commonly occurs in multiple body sites [13]. Widespread pain, a sub-group of chronic pain and the cardinal symptom of fibromyalgia is linked with a greater impact than that of pain that is not widespread [3]. This review focusses on chronic pain, but additionally examined the relationship between chronic widespread pain and mortality. Chronic widespread pain is a phenotype that captures people with more severe pain that has a greater impact on outcomes [14], [15]. We hypothesised that if chronic pain was associated with mortality that relationship would be strongest in those with chronic widespread pain.

To determine if chronic pain was associated with mortality, a systematic review was undertaken. The aims of the review were to identify and evaluate evidence to determine the strength of association between chronic pain and mortality, and in particular in the subgroup of studies investigating chronic widespread pain.

## Methods

A protocol for the conduct of this systematic review and meta-analysis was developed with reference to Centre for Reviews and Dissemination (CRD) guidelines [16] and consisted of four phases:

### Phase 1: Search strategy and identification of studies

A comprehensive search strategy was conducted by a single observer (DS). Broad search terms were used to maximise the identification of all observational studies that have examined the link between mortality and chronic and/or widespread pain. The search strategy used subject headings (e.g. MeSH where possible) and text words for death (e.g. mortality, death, survival), pain (e.g. musculoskeletal pain, fibromyalgia, joint pain) and study type (e.g. cohort studies, longitudinal studies and cross-sectional studies) (see Appendix S1 for full search strategy). The following databases were searched in March 2012: Ageline, AMED, CINAHL, EMBASE, MEDLINE, PSYCHINFO, Social Sciences Citation Index (SSCI) and Science Citation Index Expanded (SCI-EXPANDED) using ISI Web of Science. The Cochrane databases (Cochrane Database of Systematic Reviews (Cochrane Reviews) and the Database of Abstracts of Reviews of Effects (Other Reviews) (DARE) were searched for relevant reviews. Citation searches and reference list searches were undertaken to identify other possible relevant studies. A search of the Open Grey database for grey literature [17] was undertaken in April 2012 to identify any relevant papers. Searches in AMED, CINAHL, EMBASE, MEDLINE and PSYCHINFO were updated until March 2014.

### Phase 2: Study Selection

Identified studies were initially filtered with a title search by a single observer (DS) based on the following inclusion criteria:

- Study type - observational studies
- Participants – community dwelling adults
- Exposure – chronic (>3months) or widespread pain including fibromyalgia
- Outcome - mortality
- Papers published in English

A review of abstracts and keywords was then undertaken by two reviewers (DS) (RW) before the retrieval of full text articles for further screening. Disagreements were discussed during a consensus meeting with a third reviewer (JM) for final selection of studies to be included in the review.

### Phase 3: Data Extraction and Quality Assessment

From each included study, data on study population, follow-up period, pain phenotype, outcome, cause of death and potential confounding factors, were extracted by one reviewer (DS) and checked by two others (RW) (JM) for omissions and accuracy (Table 1). Two reviewers (DS, RW) then used the Quality in Prognosis Studies tool (QUIPs) [18] to assess for selection, non-response and information bias and confounding in studies. A third reviewer (JJ) reviewed selected studies to ensure consistency and consensus on judgements. Agreement between the two reviewers was also assessed by actual agreement and by agreement beyond chance (unweighted Kappa) and these were interpreted as; ≤0 = poor agreement, 0.01–0.20 = slight agreement, 0.21–0.40 = fair agreement, 0.41–0.60 = moderate agreement, 0.61–0.80 = substantial agreement, and 0.81–1.00 = almost perfect agreement [19] (Table 2).

**Table 1:** Summary of studies included in the review.

Study	n	Age	% female	Location	Follow-up	Pain phenotype	All-cause mortality	Cause specific mortality (adjusted results)	Adjusted for
**Macfarlane, G.J. et** **al. (2001)**	6569	18–85	58	NW England	8yrs	Widespread pain ACR(1990) criteria	**MRR (95%CI)**1.31 (1.05–1.65)	**MRR (95%CI)** Cancer 2.07 (1.37–3.13)Cancer without prior diagnosis 2.27(1.46–3.54) Cardiovascular disease 1.12(0.78–1.61) Respiratory disease 1.01(0.57–1.79) Other diseases 0.91 (0.45–1.85)All external causes 5.21 (0.94–28.78)	age, sex, study location
**Macfarlane, G.J. et** **al. (2007)**	7182	30 and over	54	Finland	14–16yrs	Widespread pain - in at least 4 sites(face validity with ACR(1990) criteria)	**MRR (95%CI)**0.86 (0.74–1.00)	**MRR (95%CI)** Cardiovascular disease0.83 (0.68–1.02) Cancer 0.64 (0.46–0.91)Respiratory diseases 0.89 (0.54–1.49) Otherdisease related 1.39 (0.88–2.19) Non diseaserelated 1.39 (0.75–2.58)	age, gender, education, physical work stress, mental work stress, alcohol consumption, tobacco smoking, BMI
**Andersson, H.I. (2009)**	1609	25–74	50	Sweden	14 yrs	Widespread pain - in more than four painlocations including upper and lower body andaxial pain (to get close to ACR criteria)	**MRR (95%CI)**Crude 1.95 (1.26–3.03)Adjusted 1.09 (0.62–1.90)	**MRR (95%CI)** Cardiovascular disease 2.17(1.12–4.21) Cancer 1.15 (0.52–2.55)Other 1.18 (0.47–2.99)	Age, sex, living alone, contact with friends, club membership, chronic disease, smoking, physical activity, perception of stress, BMI, insomnia (cause specific results adjusted for age and sex)
**McBeth, J. et** **al. (2009)**	4515	16 and over	51.6	NW England	8.2yrs	Widespread pain ACR (1990) criteria.Number of pain sites	**MRR (95%CI)** Crude2.4 (1.9–2.9) Adjusted1.3 (1.1–1.5)	**MRR (95%CI)** Cancer 1.8 (1.3–2.6)Cardiovascular disease 1.3 (0.99–1.6)Respiratory disease 1.0 (0.7–1.6)All external causes 0.6 (0.1–3.8)Other 0.8 (0.5–1.4)	age, sex, practice, ethic group, Townsend score of deprivation
**Sjøgren P. et** **al. (2010)**	2242	16 and over	51.3	Denmark	8 years	Chronic pain (6 months or more)	**MRR (95%CI)**Adjusted 1.21 (1.02–1.44)		age, sex, education, marital status, BMI, smoking, antidepressant use, anxiolytic use, self-reported circulatory diseases, infectious or parasitic diseases, diabetes and mental disorders
**Torrance N. et** **al. (2010)**	5853	Mean 58.43	52.7	NE Scotland	10 years	Chronic pain (more than 3 months)	**MRR (99%CI)**Crude 1.32 (1.14–1.54)Adjusted 0.90 (0.74–1.07)	**MRR (99% CI)** All circulatory system0.86 (0.65–1.14) Acute MI 1.11 (0.67–1.83)Ischaemic heart disease 1.04 (0.51–2.12)Cerebrovascular disease 0.58 (0.35–0.97)Other circulatory system 0.88 (0.47–1.66)All neoplasms 0.91 (0.64–1.28)Digestive organ neoplasms 0.97 (0.50–1.87)Respiratory organ neoplasms 0.81 (0.43–1.54)Other malignant neoplasms 0.95 (0.58–1.59)All respiratory diseases 1.23 (0.67–2.25)Pneumonia 1.44 (0.57–3.61) Chronic lowerrespiratory disease 1.35 (0.49–3.73) Otherrespiratory disease 1.08 (0.29–4.04)Diseases of the digestive system 0.90(0.35–2.34) Diseases of the nervous system0.42 (0.14–1.26) Other 0.96 (0.57–1.62)	age, sex, education, housing, long term limiting illness
**Nitter A.K. & Forseth K.Ø. (2013)**	2038	20–68 years	100	Arendal, Norway	18 years	Chronic widespread pain (in musclesand joints and back or whole body for3 months or longer)	**MRR (95%CI)**2.8 (1.3–6.1)		age, sleep problems, feeling anxious, frightened or nervous, number of non-specific health complaints
**Dreyer, L. et** **al. (2010)**	1353	19 and over	94	Denmark	15 yrs (Mean 3.9 years)	ACR (1990) definition of FM	**SMR (95%CI)**1.25 (0.9–1.7)	**SMR (95%CI) Female only:** Ischemic heartdisease 0.3 (0.0–1.6). Other heart disease3.0 (0.6–8.9) Cerebrovascular disease 3.1(1.1–6.8) Cancer 0.6 (0.3–1.2)Pneumonia 2.7 (0.0–14.8), COPD 2.0 (0.5–5.2)Liver cirrhosis 6.4 (2.3–13.9) Mental disorders2.3 (0.0–12.6) Suicide 10.5 (4.5–20.7)Other external causes 3.9 (0.1–21.7)Other 0.4 (0.1–1.5)	Standardised to Danish population (according to age, sex, calendar month)
**Wolfe, F. et** **al. (2011)**	8186	Mean 50.5, (SD 12.4)	94	USA	35yrs (Mean 7.3 years)	Fibromyalgianess scale. Widespreadpain index. ACR definition of FM1990, 2010.	**SMR (95%CI)**0.90 (0.61–1.26)	**SMR (95%CI)** Heart diseases 0.84(0.68–1.04) Cancer 0.95 (0.76–1.18)Accidents 1.45 (1.02–2.06) Chronic lowerrespiratory diseases 1.09 (0.74–1.62)Influenza and pneumonia 1.69 (1.12–2.57)Septicaemia 2.49 (1.61–3.68) Suicide 3.31(2.15–5.11) Cerebrovascular diseases 0.75(0.48–1.17) Nephritis/nephroticsyndrome/nephrosis 0.93 (0.50–1.72)Alzheimer’s disease 0.57 (0.29–1.13)Essential hypertension/hypertensive renaldisease 0.95 (0.40–2.23) Chronic liverdisease and cirrhosis 0.47 (0.16–1.38)Parkinson’s disease 0.22 (0.00–1.23)Assault (homicide) 0.26 (0.00–1.51)	Standardised to U.S. population (according to age, sex, calendar month)
**Smith, B.H. et** **al. (2003)**	10073	42–81 years	100	UK wide	6 years	Chronic pain (more than 3 months)	**AOR (95%CI)**1.1 (0.81–1.26)	**AOR (95%CI)** All cancers 0.85 (0.62–1.18)Cardiovascular disease 0.95 (0.63–1.44)Respiratory disease 2.22 (1.12–4.39) Otherdiseases 1.08 (0.52–2.27) All external causes0.99 (0.16–5.93)	age, social class, smoking

**Table 2:** Summary of agreed level of bias and percentage agreement for each potential area of bias and overall Kappa for each study.

Study	Participation		Non-response		FactorMeasurement		OutcomeMeasurement		Confounding Measurementand Account		Analysis		Overall	OverallKappa (95% CI)
	%agreement	Agreedlevel of bias	%agreement	Agreed levelof bias	%agreement	Agreed levelof bias	%agreement	Agreed levelof bias	% agreement	Agreed levelof bias	% agreement	Agreedlevel of bias	% agreement	
**Macfarlane,** **G.J., et** **al. (2001)**	67%	Moderate	100%	Moderate	100%	Low	100%	Low	88%	High	80%	Low	89%	0.85* (0.70, 0.99)
**Smith B.H.** **et** **al., (2003)**	100%	Moderate	100%	Moderate	100%	Low	100%	Low	88%	Moderate	80%	High	94%	0.86* (0.73, 0.99)
**Macfarlane,** **G.J. et** **al. (2007)**	83%	Low	67%	Moderate	100%	Low	100%	Low	88%	Moderate	60%	Moderate	83%	0.73* (0.54, 0.92)
**Andersson,** **H.I. (2009)**	67%	Moderate	83%	Moderate	100%	Low	100%	Low	100%	Low	100%	Low	95%	0.85* (0.70, 1.00)
**McBeth, J.,** **et** **al. (2009)**	100%	Low	50%	Moderate	100%	Low	100%	Low	100%	Moderate	80%	Low	89%	0.79* (0.62, 0.97)
**Dreyer, L.,** **et** **al. (2010)**	83%	Low	67%	Low	86%	Low	75%	Low	75%	High	80%	Moderate	78%	0.66* (0.47, 0.86)
**Sjogren,** **P. et** **al. (2010)**	83%	Moderate	67%	Moderate	100%	Moderate	100%	Low	63%	Low	100%	Low	83%	0.69* (0.48, 0.89)
**Torrance,** **N. et** **al. (2010)**	100%	Moderate	83%	Moderate	100%	Low	100%	Low	100%	Low	100%	Low	97%	0.96* (0.88, 1.04)
**Wolfe,** **F., et** **al. (2011)**	100%	Low	100%	Low	100%	Low	100%	Moderate	88%	Moderate	80%	Low	92%	0.88* (0.74, 1.00)
**Nitter,** **A.K. et** **al. (2013)**	100%	Moderate	100%	Moderate	86%	Moderate	75%	Moderate	75%	Moderate	60%	Moderate	83%	0.80* (0.64, 0.97)

*p<.001.

### Phase 4: Data Synthesis and Analysis

A meta-analysis was conducted to quantify heterogeneity using the I^2^ statistic and considered low if less than 25%, moderate if between 25% and 75% and high if over 75% [20]. To allow for expected heterogeneity between studies a random effects model [21] was used which applies less weight to large studies than fixed effects models [22]. Variation in the definition of chronic pain (pain phenotype) was expected to be a main source of heterogeneity and was therefore explored in a sensitivity analysis, only including studies using the stricter definition of widespread pain. A narrative review was undertaken to explore differences and similarities between included studies for age and gender, follow-up time, pain phenotype, population characteristics, methods of analysis and potential confounding factors included. Sources of heterogeneity are presented and linked to the study findings descriptively and in tabular form (Table 3).

**Table 3:** Summary of main sources of heterogeneity.

Study	Pain phenotype	Age	Location	Genderdistribution(% female)	Follow-up	Outcome (all-cause mortality)	Factors adjusted for
Macfarlane,G.J. et al. (2001)	WP	18–85years	NWEngland	58%	8yrs	**MRR (95%CI)** Adjusted1.31 (1.05–1.65)	age, sex, study location
Macfarlane,G.J. et al. (2007)	WP	30 yearsand over	Finland	54%	14–16yrs	**MRR (95%CI)** Adjusted0.86 (0.74–1.00)	age, gender, education, physical work stress,mental work stress, alcohol consumption,tobacco smoking, BMI
Andersson,H.I. (2009)	WP	25–74years	Sweden	50%	14 yrs	**MRR (95%CI)** Crude 1.95(1.26–3.03) Adjusted1.09 (0.62–1.90)	Age, sex, living alone, contact with friends,club membership, chronic disease, smoking,physical activity, perception of stress, BMI,insomnia
McBeth,J. et al. (2009)	WP	16 yearsand over	NWEngland	51.6%	8.2yrs	**MRR (95%CI)** Crude 2.4(1.9–2.9) Adjusted 1.3 (1.1–1.5)	age, sex, practice, ethic group, Townsendscore of deprivation
Sjøgren P.et al. (2010)	CP	16 yearsand over	Denmark	51.3%	8 yrs	**MRR (95%CI)** Adjusted1.21 (1.02–1.44)	age, sex, education, marital status, BMI,smoking, antidepressant use, anxiolytic use,self-reported circulatory diseases, infectiousor parasitic diseases, diabetes and mentaldisorders
Torrance N.et al. (2010)	CP	Mean 58.43years	NEScotland	52.7%	10 yrs	**MRR (95%CI)** Crude 1.32(1.14–1.54) Adjusted0.90 (0.74–1.07)	age, sex, education, housing, long term limiting illness
Nitter A.K.& Forseth K.Ø. (2013)	CWP	20–68years	Arendal,Norway	100%	18 yrs	**MRR (95%CI)** Adjusted2.8 (1.3–6.1)	age, sleep problems, feeling anxious,frightened or nervous, number ofnon-specific health complaints
Dreyer, L.et al. (2010)	FM	19 yearsand over	Denmark	94%	15 yrs (Mean 3.9 years)	**SMR (95%CI)** 1.25 (0.9–1.7)	Standardised to Danish population(according to age, sex, calendar month)
Wolfe, F.et al. (2011)	FM	Mean 50.5 years,(SD 12.4)	USA	94%	35yrs (Mean 7.3 years)	**SMR (95%CI)** 0.90 (0.61–1.26)	Standardised to U.S. population(according to age, sex, calendar month)
Smith, B.H.et al. (2003)	CP	42–81years	UK	100%	6 years	**AOR (95%CI)** 1.1 (0.81–1.26)	age, social class, smoking

Crude results were not available for all studies so the meta-analysis used the maximally adjusted results for each study. Two of the studies used standardised mortality ratios (SMRs) as their measures of effect [23], [24] and one used adjusted odds ratios (AORs) [10]. These are not directly comparable to mortality rate ratios (MRRs) so results from these studies were not included in the calculation of pooled estimates.

## Results

### Identification of studies

**Figure 1:**
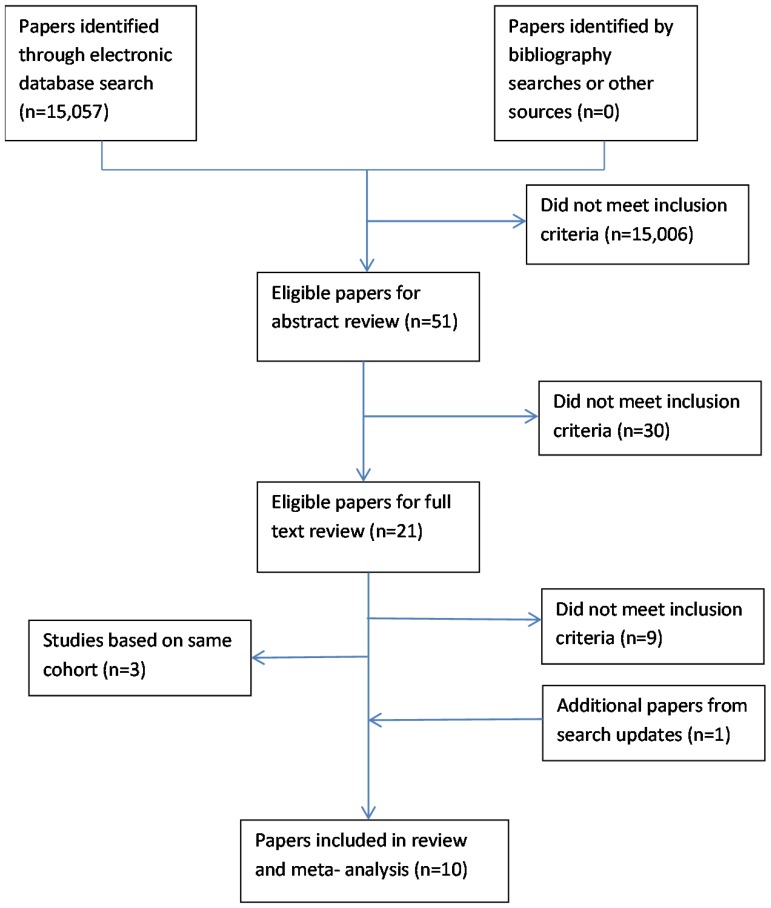
Literature search and selection.

The search identified 15,057 articles. 15,006 were excluded during the review of titles as they did not meet the inclusion criteria (Figure 1). The review of abstracts and keywords resulted in the exclusion of a further 30 articles. The full texts of 21 articles were retrieved for further screening and 9 of these were excluded; for 6 of these studies it was not possible to determine the presence of widespread pain or chronic pain lasting beyond 3 months [7], [8], [25]–[28], one study was focussed on inpatients in a pain management clinic [29], one study focussed on disease incidence as the outcome measure [30] and one examined the relationship between lifestyle factors and chronic widespread pain [31]. Twelve articles describing nine studies remained; three articles were excluded to avoid using results from the same cohort multiple times in the analysis [32]–[34] leaving nine studies for the analysis. An additional study was added from the automated database search updates in 2013 [35] resulting in 10 studies for the analysis. There was wide variation between included studies in terms of pain phenotype, follow-up time, population characteristics and inclusion of confounders (Table 1).

#### Data quality

All ten studies were of good methodological quality. Reviewers agreed the risk of selection, measurement and non-response bias was low and the greatest risk of potential bias was due to deficiencies in inclusion of putative confounders. Substantial or almost perfect agreement between reviewers regarding the level of bias was reached for all studies (Kappa statistic range 0.66 (95%CI 0.47, 0.86) to 0.96 (95%CI 0.88–1.04) (Table 2).

#### Study findings

Based on crude, unadjusted analyses one study reported chronic pain to be associated with mortality MRR 1.32 (95%CI 1.14–1.54) [36] and two studies reported that widespread pain was associated with all-cause mortality; MRR 2.4 (95%CI 1.9–2.9) [9] and MRR 1.95 (95%CI 1.26–3.03) [37]. In the first of these studies the association between chronic pain and mortality was no longer significant following adjustment for age and sex (MRR 1.15, 95%CI 0.97–1.35) [12]. In the second of these studies the association remained significant following adjustment for age, sex, medical practice, ethnic group and Townsend score of deprivation (MRR 1.3 95%CI 1.1–1.5) [9]. In the third study, the association remained significant after adjustment for age and sex (MRR 1.54, 95%CI 1.01–2.35) but was not significant following adjustment for living alone, contact with friends, club membership, comorbidity, smoking, physical activity, BMI, perception of stress and insomnia (MRR 1.09, 95%CI 0.62–1.90) [37]. Macfarlane et al., (2001) did not report crude results but did report a significant association between widespread pain and all cause-mortality following adjustment for age, sex and study location (MRR 1.31, 95%CI 1.05–1.65) [38]. In adjusted analyses Sjøgren et al., (2010) reported a significant association between chronic pain and mortality (MRR 1.21, 95%CI 1.02–1.44) [39] and where pain was determined to be both chronic and widespread Nitter and Forseth (2013) reported a greater increased risk of mortality (MRR 2.80, 95%CI 1.3–6.1). Dreyer et al (2010) reported borderline significantly increased mortality in participants with fibromyalgia (SMR 1.25 (95%CI 0.9–1.7) [23]. The remaining three studies did not report significant or strong associations between chronic or widespread pain and all-cause mortality [10], [24], [40].

### Evidence synthesis: meta-analysis

**Figure 2:**
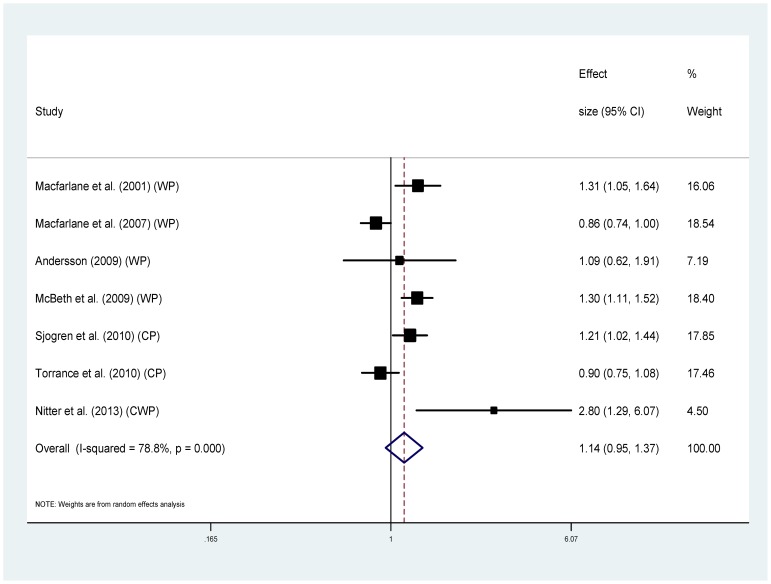
Chronic pain and all-cause mortality (using studies that reported MRR only).

**Figure 3:**
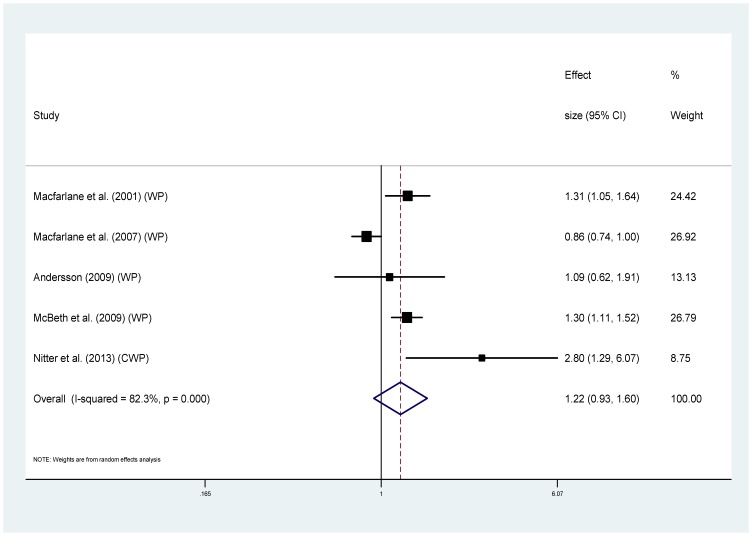
Chronic widespread pain and all-cause mortality (using studies that reported MRR only).

Seven of the ten studies used the same outcome measure (MRR) and were combined to give pooled estimates. For the association between chronic pain and all-cause mortality, the analysis showed high statistical heterogeneity (I^2^ = 78.8%) [20], with MRRs ranging from 0.86 to 2.80. The pooled estimate was small and not significant; MRR 1.14 (95%CI 0.95–1.37) (p = 0.162) (Figure 2). The sensitivity analysis including only studies measuring widespread pain indicated a slightly higher risk of mortality although the association was not significant and heterogeneity remained high (MRR range 0.86–2.8, I^2^ 82.3%, pooled MRR 1.22 (95%CI 0.93–1.60) (p = 0.157)) (Figure 3).

**Figure 4:**
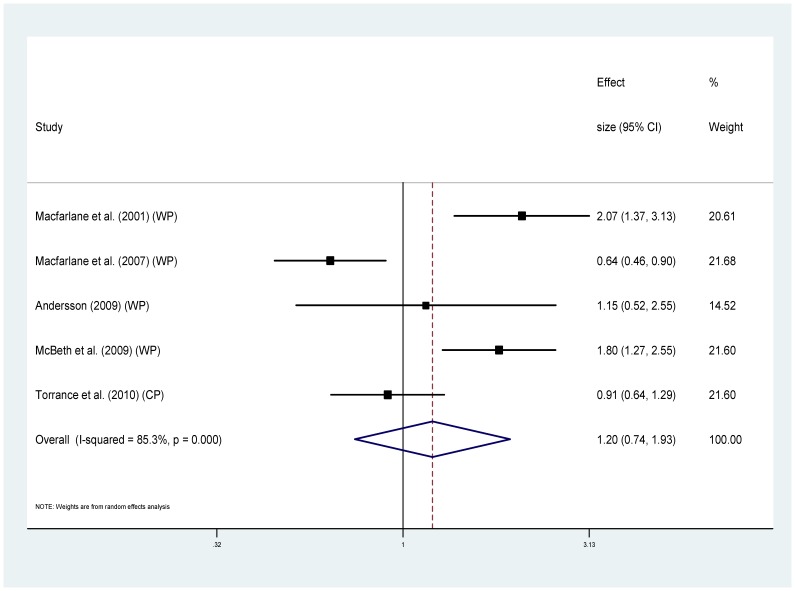
Chronic pain and cancer deaths.

**Figure 5:**
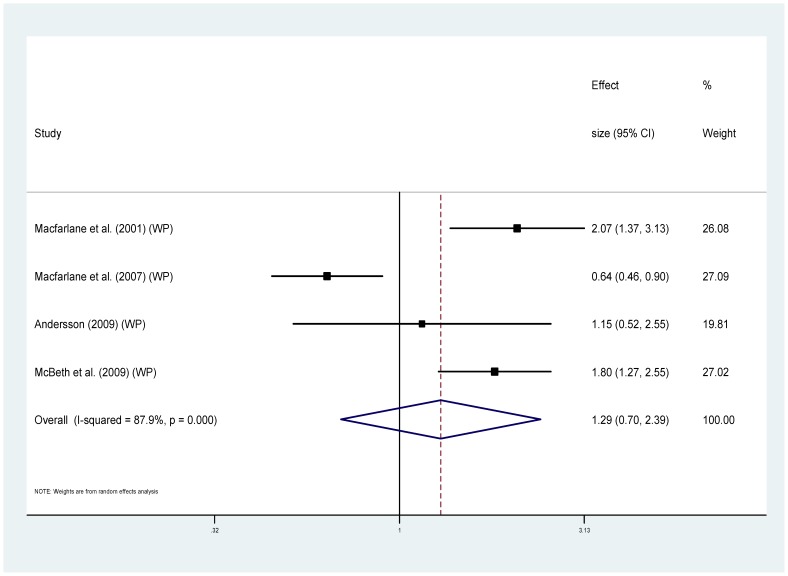
Chronic widespread pain and cancer deaths.

**Figure 6:**
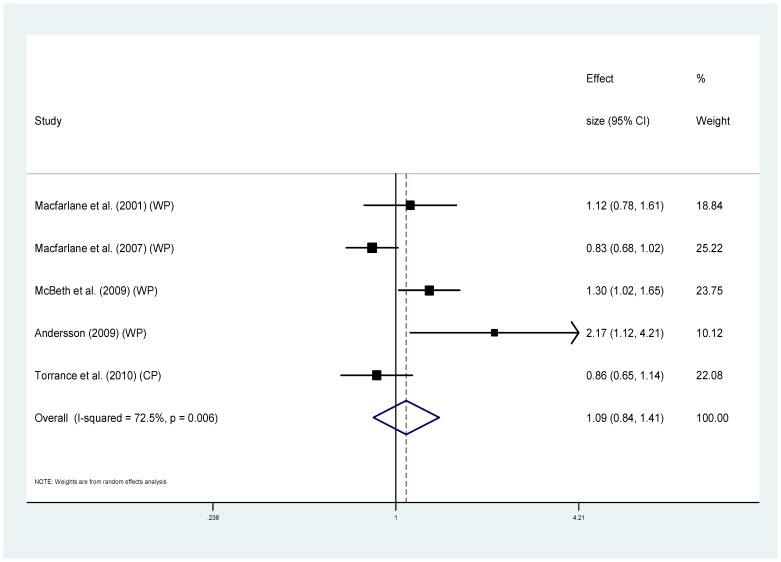
Chronic pain and cardiovascular disease deaths.

**Figure 7:**
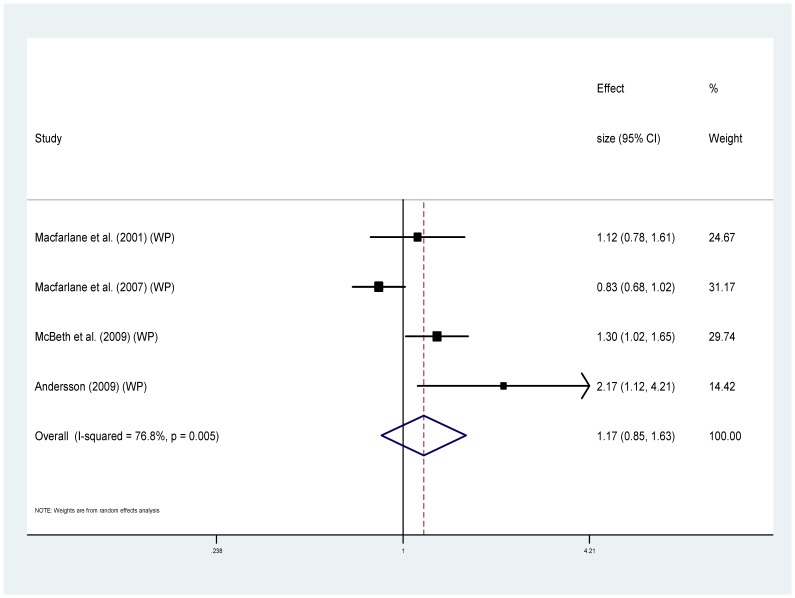
Chronic widespread pain and cardiovascular disease deaths.

**Figure 8:**
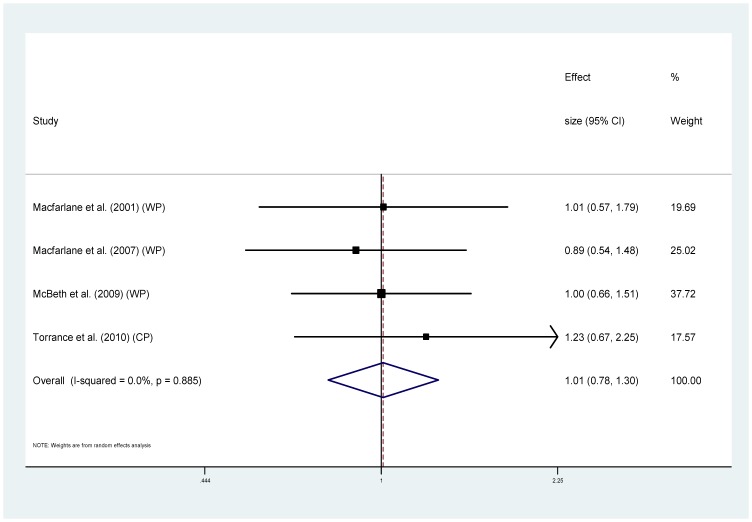
Chronic pain and respiratory disease deaths.

**Figure 9:**
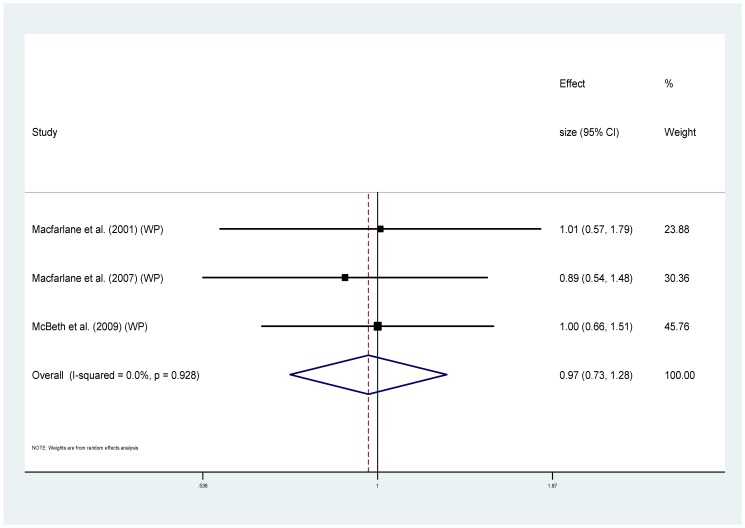
Chronic widespread pain and respiratory disease deaths.

Eight of the studies provided information regarding cause specific mortality [9], [10], [23], [24], [36]–[38], [40] (Table 1). Five of these used MRRs and could be combined to provide pooled estimates. For cancer (Figure 4), the MRRs ranged from 0.64 to 2.07 (I^2^ = 85.3%), pooled estimate MRR1.20 (95% CI 0.74–1.93) (p = 0.459). For the sub-group of studies measuring widespread pain and cancer mortality (Figure 5) MRRs ranged from 0.64–2.07 (I^2^ = 87.9%), pooled estimate MRR 1.29 (95% CI 0.70–2.39) (p = 0.417). For cardiovascular disease mortality (Figure 6), the MRRs ranged from 0.83 to 2.17 (I^2^ = 72.5%) pooled estimate MRR 1.09 (95%CI 0.84–1.41) (p = 0.536). In the widespread pain subgroup (Figure 7) the MRRs ranged from 0.83 to 2.17 (I^2^ = 76.8%), pooled estimate MRR 1.17 (95% CI 0.85–1.63) (p = 0.338). Only four studies provided information about respiratory disease mortality (Figure 8). The effect sizes ranged from 0.89 to 1.23 (I^2^ = 0.0%), pooled estimate MRR 1.01, (95%CI 0.78 to 1.30) (p = 0.944). For the widespread pain subgroup (Figure 9) the MRRs ranged from 0.89 to 1.01 (I^2^ = 0.0%), pooled estimate MRR 0.97 (95% CI 0.73–1.28) (p = 0.817). Of the studies not included in the meta-analysis, the only study reporting an increased risk of mortality from one of these three main causes of death was Smith et al., (2003) who reported an increased risk of respiratory disease mortality for women with chronic pain (AOR 2.22, 95%CI 1.1–4.39).

### Evidence synthesis: narrative review of potential sources of heterogeneity

The high heterogeneity between studies may be explained by the small number of included studies and differences in the age and gender distribution of study samples, follow-up time, pain phenotype, population characteristics, methods of analysis and potential confounding factors included (Table 3).

#### Age and gender distribution

The age and gender distribution of the study samples varied between studies but there were no clear differences in the results that could be attributed to this. The meta-analysis was conducted using results which were adjusted for age and gender limiting the influence of these factors on the observed heterogeneity.

#### Follow up time

Follow up time varied between studies (see Table 3). The shortest was 6 years [10] and in the longest participants were followed for up to 35 years [24] but this was not clearly associated with the differences in findings between studies.

#### Pain phenotype

All studies measuring widespread pain reported how closely their phenotype adhered to the criteria proposed by the American College of Rheumatology (ACR) in 1990, [41] however there were inconsistencies between studies (see Table 1). The least stringent definition of widespread pain was applied by Macfarlane et al (2007) who did not find an association between widespread pain and mortality [40]. In contrast, the three studies that defined widespread pain similar to the ACR 1990 definition [9], [37], [38] did report an association between widespread pain and mortality. Nitter and Forseth (2013) who were able to determine chronic widespread pain in all participants reported the greatest increased risk of mortality in adjusted analyses [35]. Differences in pain phenotype were explored in the sensitivity analysis in the meta-analysis, where a slight increase in the pooled estimate was observed when the analysis was restricted to studies measuring pain that was widespread.

#### Population characteristics

The study setting is likely to explain some of the heterogeneity. Four of the studies were carried out in the UK [9], [10], [36], [38], two in Denmark [23], [39], one in Finland [40], one in Sweden [37], one in Norway [35] and one in the USA [24]. A greater proportion of the participants in the Macfarlane et al., (2007) study lived in rural settings [40] compared to the Macfarlane et al., (2001) and the McBeth et al., (2009) studies which although different cohorts, were both carried out in the same urban area of the UK [9], [38]. These two studies in the urban area reported an increased risk of mortality for people with widespread pain and an increased risk of cancer mortality which were not supported by the study in a rural setting [40].

Eight of the studies were carried out in population cohorts [9], [10], [35]–[40] and two were clinical cohorts [23], [24]. Both clinical cohorts reported no significant increased risk of mortality for fibromyalgia patients.

#### Inclusion of confounders

There was wide variability in the type and number of factors adjusted for between studies. Only three of the studies reported crude results [9], [36], [37], all indicating a significant association between chronic or widespread pain and mortality. It was difficult to determine a clear pattern of how differences in the factors adjusted for determined variability in the findings between the studies. Andersson (2009) measured and adjusted for the highest number of potentially confounding factors (see Table 3 for details) and concluded their observed increased risk of mortality for people with widespread pain could be explained by lifestyle factors such as smoking and physical activities together with reported stress and disturbed sleep [37]. McBeth et al., (2009), and Nitter and Forseth (2013) did not include any lifestyle factors in their analyses and the increased risk of mortality observed in these studies was robust to adjustment of the factors they included [9], [35]. However, Torrance et al., (2010) reported the association between chronic pain and mortality attenuated to non-significance following adjustment without including lifestyle factors [36] (Table 3).

## Discussion

### Summary of findings

The results of this systematic review and meta-analysis suggest that there is a modest relationship between chronic pain and increased mortality but this is not significant. The results also suggest this relationship may be explained by cancer mortality. Confidence in these findings is restricted due to the small number of included studies and heterogeneity between them.

Restricting the analysis to studies measuring widespread pain and mortality resulted in an increase in the size of the pooled estimates for all-cause, cancer and cardiovascular disease mortality but these remained non-significant. Very few studies have examined the relationship between chronic pain and mortality. Only three studies reported crude results, all of which suggested there was an association between chronic or widespread pain and an increased rate of all-cause mortality [9], [36], [37]. However adjustment for confounders led to attenuation of the relationship. This suggests that adults with chronic or widespread pain have an increased mortality rate and this is explained by socio-demographic and lifestyle factors, however the low number of studies and high heterogeneity reduces the certainty of this. Significant associations between chronic or widespread pain and increased risk of death from cancer, cardiovascular disease, cerebrovascular disease, liver cirrhosis, suicide, accidents, influenza and pneumonia, septicaemia were reported in single studies.

### Differences between studies

The differences between the ten studies led to high levels of heterogeneity. The study populations differed on a number of characteristics which will have contributed to the variance in prevalence rates of widespread pain and mortality, and the relationship between them.

#### Definition of chronic or widespread pain

Information regarding the location of pain was lacking in three of the included studies so the presence of widespread pain could not be confirmed although chronic pain often occurs in multiple sites [13]. Similarly, details regarding the chronicity of pain were not available in all studies measuring widespread pain but in 80–90% of persons reporting widespread pain, the pain has been present for more than three months [38]. Although this means a small proportion of participants may have been misclassified it would also lead to an underestimation of the true effect. More rigorous definitions of widespread pain were more strongly associated with mortality. In additional analysis, Wolfe et al. (2011) reported that within those with fibromyalgia, those satisfying the more stringent ACR 2010 criteria [42] had an increased risk of mortality than those who did not meet the new criteria but met the 1990 criteria (HR1.62, 95%CI 1.19–2.21) [24]. The ACR 2010 criteria extend the scope of defining widespread pain beyond the location of pain by including an assessment of the severity of accompanying symptoms [43]. Increasing severity and duration of pain, in addition to extent, increases the risk of mortality [36], [44]. The revised ACR 2010 criteria, which can be measured using self-report tools in epidemiological and clinical studies, offers the potential to harmonise definitions of widespread pain in future studies [43].

#### Methods of analysis

There were differences in the referent group between studies which prevented clear comparisons. Eight studies used participants with no pain as the referent group and two used standardised populations, one in Denmark [23] and one in the US [24] which include people both with and without pain. In an additional analysis, Wolfe and colleagues (2011) compared mortality rates between fibromyalgia and osteoarthritis patients and reported no significant difference [24]. Mortality risk is increased in those with osteoarthritis compared to the general population [45] therefore this finding is not comparable to the mortality rate ratios used in other studies. The comparison group (osteoarthritis patients) cannot be considered analogous to a no pain group.

Different methods of analysis were used to calculate the outcome measures (SMR, MRR and AOR) due to variations in referent groups. Sufficient data were not available to enable conversion to comparable outcome measures for all studies. The effects of these different analysis techniques is demonstrated by McBeth et al (2009), who reported a 30% increased risk of mortality for people with widespread pain compared to a no pain group but when compared to the mortality rate for North West England mortality risk was lower and not significant (SMR = 1.14 95%CI 0.99–1.30) [9]. The more similar the referent group, the less likely a relationship will be observed. Use of general population cohorts in which a clear no-pain group can be identified will allow a purer examination of the link of increased mortality for those with widespread pain as it would enable comparison between a group most at risk (those with widespread pain) with those least at risk (those with no pain).

#### Follow-up time

Variations in follow-up time between studies may influence mortality rates although this was not clear from this review. The mortality rate in those with pain has been shown to be higher in the earlier periods of follow-up [8]. Jordan et al., (2013) have also demonstrated the strength of associations between pain in different musculoskeletal sites and cancer diminished with time indicating pain may be a marker of rather than a cause of cancer [46]. However, McBeth et al., (2009) reported no change in the relationship between widespread pain and both cancer and cardiovascular death after excluding participants who had died in the first year of follow up [9]. Standard periods of follow-up, allowing both short and long term assessment of risk would enable comparisons between studies and allow a more accurate picture of the relationship to be determined.

#### Mechanisms

Differences in study setting may contribute to variations in factors associated with the presence of pain and mortality. Pain experienced by those in rural settings may be more likely to be related to physical labour than those in urban settings [9], although this may mean they are more physically active. Physical activity is known to reduce the risk of chronic diseases and premature death [47]. Clinical and population cohorts will also have different characteristics of pain, health and socio-demographic factors. Depending on how healthcare is accessed, the clinical cohorts may have more severe symptoms and comorbidities compared to general population samples, which will impact on mechanisms to and rates of mortality. Wolfe and colleagues (2011) report that the fibromyalgia patients in their study may have higher socioeconomic status than the general population as the majority had medical insurance and received care from specialists rather than general physicians [24]. Higher socioeconomic status and access to care are associated with survival and may explain why no relationship between fibromyalgia and mortality was found in these studies [48].

The studies included in this review treated covariates as confounders and adjusted for them in their analyses. Information regarding the relative contribution of individual confounders to the models was not available. However, it may be that these factors are instead moderating or mediating the relationship between pain and mortality. Simply adjusting for such factors may lead to spurious associations between predictor and outcome [49]. Adjustment for confounders in the ten studies, such as age, gender and socio-demographic status, indicated that they had a role in the relationship between widespread pain and mortality. Comparing similarly adjusted results will control for some of the variance in the associations between studies due to different population characteristics (e.g. differences in age and gender distributions). However differences in how these factors were measured and classified will have contributed to the heterogeneity. For example, one study used the Townsend score of deprivation as a measure of socioeconomic status; this is an area-level measure which is derived from variables representing unemployment, overcrowding within households, non-home ownership and lack of car ownership [9]. In contrast the other studies included individual level measures of socio-economic status, such as educational attainment and owning medical insurance [24].

There were substantial differences in the number of additional factors measured and adjusted for between studies (Table 3). Notably the significant relationship reported in the crude analysis by Andersson and colleagues (2009) attenuated and was no longer significant when adjusted for living alone, contact with friends, club membership, comorbidity, smoking, physical activity, BMI, perception of stress and insomnia [37] indicating possible pathways between pain and mortality. Pain is associated with depression, obesity, a reduction in physical activity [50] and smoking motivation [51]. Wolfe et al., (2011) found BMI and smoking to be significant predictors of mortality in a sub-section of participants [24]. Many of the diseases where increased mortality was observed have links to lifestyle factors. For example, cancer and cardiovascular disease are associated with reduced physical activity [47] and cancer with smoking [52]. A follow up to the Macfarlane et al., (2001) study using the same cohort reported the association they observed was with both cancer incidence and survival; specifically with breast and prostate cancers [34], both of which have been shown to be associated with physical inactivity [53], [54].

Consideration of potential mechanisms between pain and mortality with the appropriate designation of mediators and moderators rather than simple adjustment for confounders would further the understanding of whether a relationship between pain and mortality exists and why. Such analysis would enable potentially modifiable characteristics to be determined that could be targeted to reduce any identified risk of mortality. There are a number of potential confounders and mediators that were not considered in the identified studies in this review which are associated with both pain and mortality, such as anxiety and depression [55], [56] social participation and social isolation [57], [58] and fatigue [59], [60].

### Strengths and Limitations

There were a number of strengths in this current study. A systematic approach was undertaken to maximise the chances of identifying all relevant studies of chronic pain and mortality. The quality of the identified studies was assessed using an established appraisal tool designed to focus on potential bias within studies [18]. Agreement between reviewers of the risk of bias was high (Table 2). Study weaknesses include: this review only included papers that were published in English and it is possible some relevant findings may have been missed. However a recent search of CINAHL, Medline and EMBASE did not find any relevant non-English studies.

The small number of studies (k<20) means that I^2^ values should be interpreted with caution as there is little power to detect true heterogeneity and as such any pooled calculation of effect may be misleading [61]. A more complex meta-analysis could have been undertaken to include the studies with multiple follow-ups and account for the correlation between them. However, even with a greater number of studies, there is general concern regarding the appropriateness of the use of meta-analyses in reviews of observational studies due to the likelihood for high heterogeneity as a result of difficulties in overcoming selection and confounding biases common to this type of study [62]. The estimates of pooled effects calculated in meta-analyses of observational studies are likely to be flawed and it is therefore more useful to have an assessment of the potential sources of heterogeneity as the focus of such systematic reviews (which has been included here) rather than a statistical combination of the data [63].

### Implications for research

This review indicated a modest association between chronic or widespread pain and increased mortality, particularly cancer mortality but further research is needed to confirm this. As stated in a commentary by Crombie (2001) even a small increased risk of cancer mortality is serious when it applies to a large proportion of the population [64]. The findings of this systematic review indicate lifestyle factors may have an important role and these may be specific to particular conditions [34]. Future research should also investigate potential mechanisms for a relationship between widespread pain and mortality directly in large population studies where specific mediators and moderators can be assessed using appropriate statistical methods. Attention should also be given to previously unmeasured social, physiological and psychological factors. The identification of pathways between pain and mortality would facilitate the identification of individuals at risk.

### Conclusions

Following this systematic review, a modest but non-significant relationship between chronic pain and mortality is suggested, particularly cancer mortality. Harmonised data collection, consistent pain phenotypes, sample populations and methods of analyses are required to robustly determine whether chronic pain increases the risk of mortality, and if so to identify the pathways by which this occurs. This combined with an investigation of the role of health, lifestyle, social and psychological factors could provide a clearer picture of the relationship between chronic pain and mortality.

## Supporting Information

Appendix S1 **Full search strategy.**
(DOCX)

Checklist S1 **PRISMA checklist.**
(DOC)
