# Supplementary material for: Chronic Pain and Mortality: A Systematic Review
Source: PLoS One. 2014 Jun 5;9(6):e99048. doi: 10.1371/journal.pone.0099048 (PMC4047043; doi:10.1371/journal.pone.0099048)
Supplement: Appendix S1 — Full search strategy. (DOCX) [file pone.0099048.s001.docx]

**Appendix S1 – Full search strategy**

**MEDLINE using OVID SP (1946 to present)**

| \| 1 \| mortality.ti,ab. \| 380773 \| \| --- \| --- \| --- \| \| 2 \| mortality/ \| 31524 \| \| 3 \| cause of death/ \| 31017 \| \| 4 \| fatal outcome/ \| 43948 \| \| 5 \| hospital mortality/ \| 17629 \| \| 6 \| "cause of death".ti,ab. \| 31705 \| \| 7 \| fatal*.ti,ab. \| 89282 \| \| 8 \| death.ti,ab. \| 389950 \| \| 9 \| death/ \| 10928 \| \| 10 \| death,sudden/ \| 10434 \| \| 11 \| dead.ti,ab. \| 32464 \| \| 12 \| died.ti,ab. \| 165667 \| \| 13 \| survival/ \| 3445 \| \| 14 \| survival.ti,ab. \| 485299 \| \| 15 \| 1 or 2 or 3 or 4 or 5 or 6 or 7 or 8 or 9 or 10 or 11 or 12 or 13 or 14 \| 1296926 \| \| 16 \| (widespread adj3 pain).ti,ab. \| 979 \| \| 17 \| arthralgia.ti,ab. \| 3547 \| \| 18 \| fibromyalgia.ti,ab. \| 5476 \| \| 19 \| myalgia.ti,ab. \| 4138 \| \| 20 \| (chronic adj3 pain).ti,ab. \| 29541 \| \| 21 \| (joint adj pain).ti,ab. \| 3430 \| \| 22 \| (radicular adj pain).ti,ab. \| 1362 \| \| 23 \| (regional adj3 pain).ti,ab. \| 2046 \| \| 24 \| "presence of pain".ti,ab. \| 630 \| \| 25 \| (mult* adj3 pain).ti,ab. \| 3132 \| \| 26 \| (comorbid* adj3 pain).ti,ab. \| 376 \| \| 27 \| musculoskeletal pain/ \| 81 \| \| 28 \| chronic pain/ \| 281 \| \| 29 \| exp joint pain/ \| 5916 \| \| 30 \| fibromyalgia/ \| 5322 \| \| 31 \| pain measurement/ \| 50607 \| \| 32 \| "non* cancer pain".ti,ab. \| 291 \| \| 33 \| "non* malignant pain".ti,ab. \| 239 \| \| 34 \| pain/mo \| 70 \| \| 35 \| arthralgia/ \| 3670 \| \| 36 \| complex regional pain syndromes/ \| 676 \| \| 37 \| myofascial pain syndromes/ \| 1079 \| \| 38 \| ((NECK or CERVICAL) adj3 PAIN).ti,ab. \| 6779 \| \| 39 \| ((KNEE* or HIP or HIPS or SHOULDER*) adj3 PAIN).ti,ab. \| 10990 \| \| 40 \| ((FOOT or FEET or ANKLE* or ELBOW*) adj3 PAIN).ti,ab. \| 2364 \| \| 41 \| ((MUSCULO* or MUSCULAR) adj3 PAIN).ti,ab. \| 3624 \| \| 42 \| ((BACK or LUMBAR or LUMBO* or SPINE or SPINAL) adj3 PAIN).ti,ab. \| 29792 \| \| 43 \| Back Pain/mo [Mortality] \| 9 \| \| 44 \| Shoulder Pain/mo [Mortality] \| 1 \| \| 45 \| 16 or 17 or 18 or 19 or 20 or 21 or 22 or 23 or 24 or 25 or 26 or 27 or 28 or 29 or 30 or 31 or 32 or 33 or 34 or 35 or 36 or 37 or 38 or 39 or 40 or 41 or 42 or 43 or 44 \| 132018 \| \| 46 \| Longitudinal Studies/ \| 68770 \| \| 47 \| Cohort Studies/ \| 129164 \| \| 48 \| Prospective Studies/ \| 310346 \| \| 49 \| Family Practice/ or General Practice/ \| 59151 \| \| 50 \| Retrospective Studies/ \| 403154 \| \| 51 \| Case-Control Studies/ or Epidemiologic Methods/ \| 170484 \| \| 52 \| Cross-Sectional Studies/ \| 134718 \| \| 53 \| "family pract*".ti,ab. \| 8324 \| \| 54 \| "general pract*".ti,ab. \| 54841 \| \| 55 \| (observ* or cohort or prospectiv* or retrospectiv* or population or longitud* or community or case* control or cross* section*).ti,ab. \| 3556111 \| \| 56 \| 46 or 47 or 48 or 49 or 50 or 51 or 52 or 53 or 54 or 55 \| 3957958 \| \| 57 \| 15 and 45 and 56 \| 2342 \| \| 58 \| limit 57 to (english language and humans) \| **1935** \| |  |  |
| --- | --- | --- | --- | --- | --- | --- | --- | --- | --- | --- | --- | --- | --- | --- | --- | --- | --- | --- | --- | --- | --- | --- | --- | --- | --- | --- | --- | --- | --- | --- | --- | --- | --- | --- | --- | --- | --- | --- | --- | --- | --- | --- | --- | --- | --- | --- | --- | --- | --- | --- | --- | --- | --- | --- | --- | --- | --- | --- | --- | --- | --- | --- | --- | --- | --- | --- | --- | --- | --- | --- | --- | --- | --- | --- | --- | --- | --- | --- | --- | --- | --- | --- | --- | --- | --- | --- | --- | --- | --- | --- | --- | --- | --- | --- | --- | --- | --- | --- | --- | --- | --- | --- | --- | --- | --- | --- | --- | --- | --- | --- | --- | --- | --- | --- | --- | --- | --- | --- | --- | --- | --- | --- | --- | --- | --- | --- | --- | --- | --- | --- | --- | --- | --- | --- | --- | --- | --- | --- | --- | --- | --- | --- | --- | --- | --- | --- | --- | --- | --- | --- | --- | --- | --- | --- | --- | --- | --- | --- | --- | --- | --- | --- | --- | --- | --- | --- | --- | --- | --- | --- | --- | --- | --- | --- | --- | --- |
|  |  |  |

**EMBASE using OVID SP (1980 to present)**

| 1 | mortality.ti,ab. | 455842 |
| --- | --- | --- |
| 2 | mortality/ | 400684 |
| 3 | cancer mortality/ | 40133 |
| 4 | standardized mortality ratio/ | 470 |
| 5 | "cause of death".ti,ab. | 38288 |
| 6 | exp cause of death/ | 54041 |
| 7 | death.ti,ab. | 454083 |
| 8 | death/ or fatality/ | 143765 |
| 9 | dead.ti,ab. | 36715 |
| 10 | sudden death/ | 30665 |
| 11 | died.ti,ab. | 191213 |
| 12 | fatal*.ti,ab. | 101502 |
| 13 | survival/ | 130224 |
| 14 | survival.ti,ab. | 571242 |
| 15 | 1 or 2 or 3 or 4 or 5 or 6 or 7 or 8 or 9 or 10 or 11 or 12 or 13 or 14 | 1668788 |
| 16 | (widespread adj3 pain).ti,ab. | 1352 |
| 17 | fibromyalgia/ | 10368 |
| 18 | chronic pain/ | 25260 |
| 19 | arthralgia/ | 28014 |
| 20 | arthralgia.ti,ab. | 4703 |
| 21 | pain assessment/ | 53996 |
| 22 | fibromyalgia.ti,ab. | 7996 |
| 23 | myalgia/ | 27190 |
| 24 | myalgia.ti,ab. | 5341 |
| 25 | (chronic adj3 pain).ti,ab. | 39396 |
| 26 | exp musculoskeletal pain/ | 3051 |
| 27 | (joint adj pain).ti,ab. | 4397 |
| 28 | radicular pain/ | 1671 |
| 29 | (regional adj3 pain).ti,ab. | 3222 |
| 30 | (radicular adj pain).ti,ab. | 1724 |
| 31 | "presence of pain".ti,ab. | 802 |
| 32 | (mult* adj3 pain).ti,ab. | 4492 |
| 33 | (comorbid* adj3 pain).ti,ab. | 520 |
| 34 | "non* cancer pain".ti,ab. | 477 |
| 35 | "non* malignant pain".ti,ab. | 375 |
| 36 | complex regional pain syndrome/ | 1807 |
| 37 | myofascial pain/ | 6245 |
| 38 | ((NECK or CERVICAL) adj3 PAIN).ti,ab. | 8353 |
| 39 | ((KNEE* or HIP or HIPS or SHOULDER*) adj3 PAIN).ti,ab. | 13404 |
| 40 | ((BACK or LUMBAR or LUMBO* or SPINE or SPINAL) adj3 PAIN).ti,ab. | 37797 |
| 41 | ((FOOT or FEET or ANKLE* or ELBOW*) adj3 PAIN).ti,ab. | 2952 |
| 42 | ((MUSCULO* or MUSCULAR) adj3 PAIN).ti,ab. | 4840 |
| 43 | hip pain/ or low back pain/ or spinal pain/ or foot pain/ or ankle pain/ or bone pain/ or knee pain/ or shoulder pain/ or neck pain/ | 59850 |
| 44 | 16 or 17 or 18 or 19 or 20 or 21 or 22 or 23 or 24 or 25 or 26 or 27 or 28 or 29 or 30 or 31 or 32 or 33 or 34 or 35 or 36 or 37 or 38 or 39 or 40 or 41 or 42 or 43 | 229400 |
| 45 | prospective study/ | 183136 |
| 46 | cohort analysis/ | 109928 |
| 47 | population research/ | 55964 |
| 48 | longitudinal study/ | 48485 |
| 49 | observational study/ | 26687 |
| 50 | community assessment/ | 1090 |
| 51 | general practice/ | 62244 |
| 52 | retrospective study/ | 251160 |
| 53 | case control study/ | 57709 |
| 54 | cross-sectional study/ | 63425 |
| 55 | "family pract*".ti,ab. | 8406 |
| 56 | "general pract*".ti,ab. | 65835 |
| 57 | (observ* or cohort or prospectiv* or retrospectiv* or population or longitud* or community or case* control or cross* section*).ti,ab. | 4024951 |
| 58 | 45 or 46 or 47 or 48 or 49 or 50 or 51 or 52 or 53 or 54 or 55 or 56 or 57 | 4238696 |
| 59 | 15 and 44 and 58 | 5684 |
| 60 | limit 59 to (human and english language) | **4651** |

**AMED using OVID SP (1985 to present)**

| \| 1 \| mortality.ti,ab. \| 1718 \| \| --- \| --- \| --- \| \| 2 \| Mortality/ \| 776 \| \| 3 \| Death/ \| 1564 \| \| 4 \| "cause of death".ti,ab. \| 235 \| \| 5 \| death.ti,ab. \| 4905 \| \| 6 \| fatal outcome/ \| 4 \| \| 7 \| Death sudden/ \| 41 \| \| 8 \| dead.ti,ab. \| 207 \| \| 9 \| died.ti,ab. \| 1176 \| \| 10 \| survival.ti,ab. \| 1982 \| \| 11 \| fatal*.ti,ab. \| 305 \| \| 12 \| 1 or 2 or 3 or 4 or 5 or 6 or 7 or 8 or 9 or 10 or 11 \| 9383 \| \| 13 \| (widespread adj3 pain).ti,ab. \| 206 \| \| 14 \| Arthralgia/ \| 100 \| \| 15 \| arthralgia.ti,ab. \| 39 \| \| 16 \| fibromyalgia/ \| 1448 \| \| 17 \| fibromyalgia.ti,ab. \| 1542 \| \| 18 \| myalgia.ti,ab. \| 61 \| \| 19 \| (chronic adj3 pain).ti,ab. \| 3521 \| \| 20 \| (joint adj pain).ti,ab. \| 195 \| \| 21 \| (radicular adj pain).ti,ab. \| 64 \| \| 22 \| complex regional pain syndromes/ \| 28 \| \| 23 \| (regional adj3 pain).ti,ab. \| 183 \| \| 24 \| "presence of pain".ti,ab. \| 52 \| \| 25 \| (mult* adj3 pain).ti,ab. \| 500 \| \| 26 \| (comorbid* adj3 pain).ti,ab. \| 41 \| \| 27 \| Pain measurement/ \| 806 \| \| 28 \| "non* cancer pain".ti,ab. \| 17 \| \| 29 \| "non* malignant pain".ti,ab. \| 21 \| \| 30 \| Myofascial pain syndromes/ \| 230 \| \| 31 \| ((NECK or CERVICAL) adj3 PAIN).ti,ab. \| 1252 \| \| 32 \| ((KNEE* or HIP or HIPS or SHOULDER*) adj3 PAIN).ti,ab. \| 1602 \| \| 33 \| ((FOOT or FEET or ANKLE* or ELBOW*) adj3 PAIN).ti,ab. \| 620 \| \| 34 \| ((BACK or LUMBAR or LUMBO* or SPINE or SPINAL) adj3 PAIN).ti,ab. \| 5461 \| \| 35 \| ((MUSCULO* or MUSCULAR) adj3 PAIN).ti,ab. \| 674 \| \| 36 \| Low back pain/ or Backache/ \| 5091 \| \| 37 \| shoulder pain/ \| 183 \| \| 38 \| Neck pain/ \| 705 \| \| 39 \| 13 or 14 or 15 or 16 or 17 or 18 or 19 or 20 or 21 or 22 or 23 or 24 or 25 or 26 or 27 or 28 or 29 or 30 or 31 or 32 or 33 or 34 or 35 or 36 or 37 or 38 \| 13771 \| \| 40 \| cohort studies/ or prospective studies/ \| 622 \| \| 41 \| Family practice/ \| 803 \| \| 42 \| epidemiologic methods/ \| 479 \| \| 43 \| "family pract*".ti,ab. \| 132 \| \| 44 \| "general pract*".ti,ab. \| 1149 \| \| 45 \| (observ* or cohort or prospectiv* or retrospectiv* or population or longitud* or community or case* control or cross* section*).ti,ab. \| 40041 \| \| 46 \| 40 or 41 or 42 or 43 or 44 or 45 \| 41539 \| \| 47 \| 12 and 39 and 46 \| **42** \| | |  | |  | |
| --- | --- | --- | --- | --- | --- | --- | --- | --- | --- | --- | --- | --- | --- | --- | --- | --- | --- | --- | --- | --- | --- | --- | --- | --- | --- | --- | --- | --- | --- | --- | --- | --- | --- | --- | --- | --- | --- | --- | --- | --- | --- | --- | --- | --- | --- | --- | --- | --- | --- | --- | --- | --- | --- | --- | --- | --- | --- | --- | --- | --- | --- | --- | --- | --- | --- | --- | --- | --- | --- | --- | --- | --- | --- | --- | --- | --- | --- | --- | --- | --- | --- | --- | --- | --- | --- | --- | --- | --- | --- | --- | --- | --- | --- | --- | --- | --- | --- | --- | --- | --- | --- | --- | --- | --- | --- | --- | --- | --- | --- | --- | --- | --- | --- | --- | --- | --- | --- | --- | --- | --- | --- | --- | --- | --- | --- | --- | --- | --- | --- | --- | --- | --- | --- | --- | --- | --- | --- | --- | --- | --- | --- | --- | --- | --- | --- | --- |
|  |  | |  | |  |

**PSYCHINFO using OVID SP (1802 to present)**

| \| \| 1 \| mortality.ti,ab. \| 19078 \| \| --- \| --- \| --- \| \| 2 \| Mortality Rate/ \| 4561 \| \| 3 \| "cause of death".ti,ab. \| 2333 \| \| 4 \| "Death and Dying"/ \| 19022 \| \| 5 \| fatal*.ti,ab. \| 6614 \| \| 6 \| death.ti,ab. \| 48028 \| \| 7 \| dead.ti,ab. \| 3838 \| \| 8 \| died.ti,ab. \| 8193 \| \| 9 \| survival.ti,ab. \| 20460 \| \| 10 \| 1 or 2 or 3 or 4 or 5 or 6 or 7 or 8 or 9 \| 95102 \| \| 11 \| (widespread adj3 pain).ti,ab. \| 286 \| \| 12 \| arthralgia.ti,ab. \| 47 \| \| 13 \| fibromyalgia.ti,ab. \| 1787 \| \| 14 \| myalgia.ti,ab. \| 150 \| \| 15 \| (chronic adj3 pain).ti,ab. \| 10512 \| \| 16 \| (joint adj pain).ti,ab. \| 264 \| \| 17 \| (radicular adj pain).ti,ab. \| 64 \| \| 18 \| (regional adj3 pain).ti,ab. \| 481 \| \| 19 \| "presence of pain".ti,ab. \| 150 \| \| 20 \| (mult* adj3 pain).ti,ab. \| 1357 \| \| 21 \| (comorbid* adj3 pain).ti,ab. \| 205 \| \| 22 \| "non* cancer pain".ti,ab. \| 103 \| \| 23 \| "non* malignant pain".ti,ab. \| 81 \| \| 24 \| Myofascial Pain/ \| 255 \| \| 25 \| ((NECK or CERVICAL) adj3 PAIN).ti,ab. \| 764 \| \| 26 \| ((KNEE* or HIP or HIPS or SHOULDER*) adj3 PAIN).ti,ab. \| 521 \| \| 27 \| ((FOOT or FEET or ANKLE* or ELBOW*) adj3 PAIN).ti,ab. \| 104 \| \| 28 \| ((BACK or LUMBAR or LUMBO* or SPINE or SPINAL) adj3 PAIN).ti,ab. \| 3806 \| \| 29 \| ((MUSCULO* or MUSCULAR) adj3 PAIN).ti,ab. \| 908 \| \| 30 \| Back Pain/ \| 2408 \| \| 31 \| Pain Measurement/ \| 914 \| \| 32 \| fibromyalgia/ \| 929 \| \| 33 \| Chronic Pain/ \| 7980 \| \| 34 \| 11 or 12 or 13 or 14 or 15 or 16 or 17 or 18 or 19 or 20 or 21 or 22 or 23 or 24 or 25 or 26 or 27 or 28 or 29 or 30 or 31 or 32 or 33 \| 18034 \| \| 35 \| prospective studies/ or longitudinal studies/ \| 15006 \| \| 36 \| cohort analysis/ \| 889 \| \| 37 \| general practitioners/ \| 3988 \| \| 38 \| Retrospective Studies/ \| 315 \| \| 39 \| "family pract*".ti,ab. \| 1935 \| \| 40 \| "general pract*".ti,ab. \| 8495 \| \| 41 \| (observ* or cohort or prospectiv* or retrospectiv* or population or longitud* or community or case* control or cross* section*).ti,ab. \| 614867 \| \| 42 \| 35 or 36 or 37 or 38 or 39 or 40 or 41 \| 627780 \| \| 43 \| 10 and 34 and 42 \| 112 \| \| 44 \| limit 43 to (human and english language) \| **100** \| \|  \|  \| \| --- \| --- \| --- \| --- \| --- \| --- \| --- \| --- \| --- \| --- \| --- \| --- \| --- \| --- \| --- \| --- \| --- \| --- \| --- \| --- \| --- \| --- \| --- \| --- \| --- \| --- \| --- \| --- \| --- \| --- \| --- \| --- \| --- \| --- \| --- \| --- \| --- \| --- \| --- \| --- \| --- \| --- \| --- \| --- \| --- \| --- \| --- \| --- \| --- \| --- \| --- \| --- \| --- \| --- \| --- \| --- \| --- \| --- \| --- \| --- \| --- \| --- \| --- \| --- \| --- \| --- \| --- \| --- \| --- \| --- \| --- \| --- \| --- \| --- \| --- \| --- \| --- \| --- \| --- \| --- \| --- \| --- \| --- \| --- \| --- \| --- \| --- \| --- \| --- \| --- \| --- \| --- \| --- \| --- \| --- \| --- \| --- \| --- \| --- \| --- \| --- \| --- \| --- \| --- \| --- \| --- \| --- \| --- \| --- \| --- \| --- \| --- \| --- \| --- \| --- \| --- \| --- \| --- \| --- \| --- \| --- \| --- \| --- \| --- \| --- \| --- \| --- \| --- \| --- \| --- \| --- \| --- \| --- \| --- \| --- \| \|  \|  \|  \| |  |  |  |  |
| --- | --- | --- | --- | --- | --- | --- | --- | --- | --- | --- | --- | --- | --- | --- | --- | --- | --- | --- | --- | --- | --- | --- | --- | --- | --- | --- | --- | --- | --- | --- | --- | --- | --- | --- | --- | --- | --- | --- | --- | --- | --- | --- | --- | --- | --- | --- | --- | --- | --- | --- | --- | --- | --- | --- | --- | --- | --- | --- | --- | --- | --- | --- | --- | --- | --- | --- | --- | --- | --- | --- | --- | --- | --- | --- | --- | --- | --- | --- | --- | --- | --- | --- | --- | --- | --- | --- | --- | --- | --- | --- | --- | --- | --- | --- | --- | --- | --- | --- | --- | --- | --- | --- | --- | --- | --- | --- | --- | --- | --- | --- | --- | --- | --- | --- | --- | --- | --- | --- | --- | --- | --- | --- | --- | --- | --- | --- | --- | --- | --- | --- | --- | --- | --- | --- | --- | --- | --- | --- | --- | --- | --- | --- |
|  |  |  |  |  |

**CINAHL using NHS interface (1980 to present)**

| \| \| 1 \| MORTALITY/ OR HOSPITAL MORTALITY/ \| [15154](http://www.library.nhs.uk/booksandjournals/advanced/search.aspx?viewAction=view&resultItem=1) \| \| --- \| --- \| --- \| \| 2 \| mortality.ti,ab \| [45926](http://www.library.nhs.uk/booksandjournals/advanced/search.aspx?viewAction=view&resultItem=2) \| \| 3 \| CAUSE OF DEATH/ \| [4018](http://www.library.nhs.uk/booksandjournals/advanced/search.aspx?viewAction=view&resultItem=3) \| \| 4 \| "cause of death".ti,ab \| [3680](http://www.library.nhs.uk/booksandjournals/advanced/search.aspx?viewAction=view&resultItem=4) \| \| 5 \| FATAL OUTCOME/ \| [2216](http://www.library.nhs.uk/booksandjournals/advanced/search.aspx?viewAction=view&resultItem=5) \| \| 6 \| fatal*.ti,ab \| [8192](http://www.library.nhs.uk/booksandjournals/advanced/search.aspx?viewAction=view&resultItem=6) \| \| 7 \| DEATH/ \| [7876](http://www.library.nhs.uk/booksandjournals/advanced/search.aspx?viewAction=view&resultItem=7) \| \| 8 \| death.ti,ab \| [41030](http://www.library.nhs.uk/booksandjournals/advanced/search.aspx?viewAction=view&resultItem=8) \| \| 9 \| dead.ti,ab \| [2172](http://www.library.nhs.uk/booksandjournals/advanced/search.aspx?viewAction=view&resultItem=9) \| \| 10 \| died.ti,ab \| [9386](http://www.library.nhs.uk/booksandjournals/advanced/search.aspx?viewAction=view&resultItem=10) \| \| 11 \| SURVIVAL/ \| [13840](http://www.library.nhs.uk/booksandjournals/advanced/search.aspx?viewAction=view&resultItem=11) \| \| 12 \| survival.ti,ab \| [29672](http://www.library.nhs.uk/booksandjournals/advanced/search.aspx?viewAction=view&resultItem=12) \| \| 13 \| 1 OR 2 OR 3 OR 4 OR 5 OR 6 OR 7 OR 8 OR 9 OR 10 OR 11 OR 12 \| [129406](http://www.library.nhs.uk/booksandjournals/advanced/search.aspx?viewAction=view&resultItem=13) \| \| 14 \| NECK PAIN/ OR CHRONIC PAIN/ OR BACK PAIN/ OR PAIN MEASUREMENT/ \| [30783](http://www.library.nhs.uk/booksandjournals/advanced/search.aspx?viewAction=view&resultItem=14) \| \| 15 \| (widespread adj3 pain).ti,ab \| [329](http://www.library.nhs.uk/booksandjournals/advanced/search.aspx?viewAction=view&resultItem=15) \| \| 16 \| ARTHRALGIA/ \| [500](http://www.library.nhs.uk/booksandjournals/advanced/search.aspx?viewAction=view&resultItem=16) \| \| 17 \| arthralgia.ti,ab \| [244](http://www.library.nhs.uk/booksandjournals/advanced/search.aspx?viewAction=view&resultItem=17) \| \| 18 \| FIBROMYALGIA/ \| [2503](http://www.library.nhs.uk/booksandjournals/advanced/search.aspx?viewAction=view&resultItem=18) \| \| 19 \| fibromyalgia.ti,ab \| [2218](http://www.library.nhs.uk/booksandjournals/advanced/search.aspx?viewAction=view&resultItem=19) \| \| 20 \| myalgia.ti,ab \| [305](http://www.library.nhs.uk/booksandjournals/advanced/search.aspx?viewAction=view&resultItem=20) \| \| 21 \| (chronic adj3 pain).ti,ab \| [9552](http://www.library.nhs.uk/booksandjournals/advanced/search.aspx?viewAction=view&resultItem=21) \| \| 22 \| (joint ADJ pain).ti,ab \| [1301](http://www.library.nhs.uk/booksandjournals/advanced/search.aspx?viewAction=view&resultItem=22) \| \| 23 \| (radicular ADJ pain).ti,ab \| [258](http://www.library.nhs.uk/booksandjournals/advanced/search.aspx?viewAction=view&resultItem=23) \| \| 24 \| COMPLEX REGIONAL PAIN SYNDROMES/ \| [398](http://www.library.nhs.uk/booksandjournals/advanced/search.aspx?viewAction=view&resultItem=24) \| \| 25 \| (regional adj3 pain).ti,ab \| [742](http://www.library.nhs.uk/booksandjournals/advanced/search.aspx?viewAction=view&resultItem=25) \| \| 26 \| "presence of pain".ti,ab \| [181](http://www.library.nhs.uk/booksandjournals/advanced/search.aspx?viewAction=view&resultItem=26) \| \| 27 \| (mult* adj3 pain).ti,ab \| [1376](http://www.library.nhs.uk/booksandjournals/advanced/search.aspx?viewAction=view&resultItem=27) \| \| 28 \| (comorbid* adj3 pain).ti,ab \| [176](http://www.library.nhs.uk/booksandjournals/advanced/search.aspx?viewAction=view&resultItem=28) \| \| 29 \| "non* cancer pain".ti,ab \| [69](http://www.library.nhs.uk/booksandjournals/advanced/search.aspx?viewAction=view&resultItem=29) \| \| 30 \| "non* malignant pain".ti,ab \| [49](http://www.library.nhs.uk/booksandjournals/advanced/search.aspx?viewAction=view&resultItem=30) \| \| 31 \| MYOFASCIAL PAIN SYNDROMES/ \| [717](http://www.library.nhs.uk/booksandjournals/advanced/search.aspx?viewAction=view&resultItem=31) \| \| 32 \| (neck OR cervical adj3 pain).ti,ab \| [12840](http://www.library.nhs.uk/booksandjournals/advanced/search.aspx?viewAction=view&resultItem=32) \| \| 33 \| (knee* OR hip OR hips OR shoulder* adj3 pain).ti,ab \| [29176](http://www.library.nhs.uk/booksandjournals/advanced/search.aspx?viewAction=view&resultItem=33) \| \| 34 \| (foot OR feet OR ankle* OR elbow* adj3 pain).ti,ab \| [19010](http://www.library.nhs.uk/booksandjournals/advanced/search.aspx?viewAction=view&resultItem=34) \| \| 35 \| (back OR lumbar OR lumbo* OR spine OR spinal adj3 pain).ti,ab \| [38544](http://www.library.nhs.uk/booksandjournals/advanced/search.aspx?viewAction=view&resultItem=35) \| \| 36 \| (musculo* OR muscular adj3 pain).ti,ab \| [8239](http://www.library.nhs.uk/booksandjournals/advanced/search.aspx?viewAction=view&resultItem=36) \| \| 37 \| PELVIC PAIN/ \| [848](http://www.library.nhs.uk/booksandjournals/advanced/search.aspx?viewAction=view&resultItem=37) \| \| 38 \| 14 OR 15 OR 16 OR 17 OR 18 OR 19 OR 20 OR 21 OR 22 OR 23 OR 24 OR 25 OR 26 OR 27 OR 28 OR 29 OR 30 OR 31 OR 32 OR 33 OR 34 OR 35 OR 36 OR 37 \| [123270](http://www.library.nhs.uk/booksandjournals/advanced/search.aspx?viewAction=view&resultItem=38) \| \| 39 \| NONEXPERIMENTAL STUDIES/ OR CASE CONTROL STUDIES/ OR PROSPECTIVE STUDIES/ \| [149517](http://www.library.nhs.uk/booksandjournals/advanced/search.aspx?viewAction=view&resultItem=39) \| \| 40 \| CONCURRENT PROSPECTIVE STUDIES/ OR PSEUDOLONGITUDINAL STUDIES/ \| [55](http://www.library.nhs.uk/booksandjournals/advanced/search.aspx?viewAction=view&resultItem=40) \| \| 41 \| RETROSPECTIVE PANEL STUDIES/ OR RETROSPECTIVE DESIGN/ \| [55085](http://www.library.nhs.uk/booksandjournals/advanced/search.aspx?viewAction=view&resultItem=41) \| \| 42 \| NONCONCURRENT PROSPECTIVE STUDIES/ OR CROSS SECTIONAL STUDIES/ \| [46988](http://www.library.nhs.uk/booksandjournals/advanced/search.aspx?viewAction=view&resultItem=42) \| \| 43 \| FAMILY PRACTICE/ \| [8926](http://www.library.nhs.uk/booksandjournals/advanced/search.aspx?viewAction=view&resultItem=43) \| \| 44 \| COMMUNITY HEALTH CENTERS/ \| [2088](http://www.library.nhs.uk/booksandjournals/advanced/search.aspx?viewAction=view&resultItem=44) \| \| 45 \| OBSERVATIONAL METHODS/ \| [7756](http://www.library.nhs.uk/booksandjournals/advanced/search.aspx?viewAction=view&resultItem=45) \| \| 46 \| EPIDEMIOLOGICAL RESEARCH/ \| [17635](http://www.library.nhs.uk/booksandjournals/advanced/search.aspx?viewAction=view&resultItem=46) \| \| 47 \| "family pract*".ti,ab \| [1499](http://www.library.nhs.uk/booksandjournals/advanced/search.aspx?viewAction=view&resultItem=47) \| \| 48 \| "general pract*".ti,ab \| [9561](http://www.library.nhs.uk/booksandjournals/advanced/search.aspx?viewAction=view&resultItem=48) \| \| 51 \| (observ* OR cohort OR prospectiv* OR retrospectiv* OR population OR longitud* OR community OR (case* AND control) OR (cross* AND section*)).ti,ab \| [336270](http://www.library.nhs.uk/booksandjournals/advanced/search.aspx?viewAction=view&resultItem=51) \| \| 52 \| 39 OR 40 OR 41 OR 42 OR 43 OR 44 OR 45 OR 46 OR 47 OR 48 OR 51 \| [461010](http://www.library.nhs.uk/booksandjournals/advanced/search.aspx?viewAction=view&resultItem=52) \| \| 53 \| 13 AND 38 AND 52 \| [3263](http://www.library.nhs.uk/booksandjournals/advanced/search.aspx?viewAction=view&resultItem=53) \| \| 54 \| 53 [Limit to: (Language English)] \| [**3232**](http://www.library.nhs.uk/booksandjournals/advanced/search.aspx?viewAction=view&resultItem=54) \| \|  \|  \| \| --- \| --- \| --- \| --- \| --- \| --- \| --- \| --- \| --- \| --- \| --- \| --- \| --- \| --- \| --- \| --- \| --- \| --- \| --- \| --- \| --- \| --- \| --- \| --- \| --- \| --- \| --- \| --- \| --- \| --- \| --- \| --- \| --- \| --- \| --- \| --- \| --- \| --- \| --- \| --- \| --- \| --- \| --- \| --- \| --- \| --- \| --- \| --- \| --- \| --- \| --- \| --- \| --- \| --- \| --- \| --- \| --- \| --- \| --- \| --- \| --- \| --- \| --- \| --- \| --- \| --- \| --- \| --- \| --- \| --- \| --- \| --- \| --- \| --- \| --- \| --- \| --- \| --- \| --- \| --- \| --- \| --- \| --- \| --- \| --- \| --- \| --- \| --- \| --- \| --- \| --- \| --- \| --- \| --- \| --- \| --- \| --- \| --- \| --- \| --- \| --- \| --- \| --- \| --- \| --- \| --- \| --- \| --- \| --- \| --- \| --- \| --- \| --- \| --- \| --- \| --- \| --- \| --- \| --- \| --- \| --- \| --- \| --- \| --- \| --- \| --- \| --- \| --- \| --- \| --- \| --- \| --- \| --- \| --- \| --- \| --- \| --- \| --- \| --- \| --- \| --- \| --- \| --- \| --- \| --- \| --- \| --- \| --- \| --- \| --- \| --- \| --- \| --- \| --- \| --- \| --- \| --- \| --- \| --- \| |  |  |  |  |
| --- | --- | --- | --- | --- | --- | --- | --- | --- | --- | --- | --- | --- | --- | --- | --- | --- | --- | --- | --- | --- | --- | --- | --- | --- | --- | --- | --- | --- | --- | --- | --- | --- | --- | --- | --- | --- | --- | --- | --- | --- | --- | --- | --- | --- | --- | --- | --- | --- | --- | --- | --- | --- | --- | --- | --- | --- | --- | --- | --- | --- | --- | --- | --- | --- | --- | --- | --- | --- | --- | --- | --- | --- | --- | --- | --- | --- | --- | --- | --- | --- | --- | --- | --- | --- | --- | --- | --- | --- | --- | --- | --- | --- | --- | --- | --- | --- | --- | --- | --- | --- | --- | --- | --- | --- | --- | --- | --- | --- | --- | --- | --- | --- | --- | --- | --- | --- | --- | --- | --- | --- | --- | --- | --- | --- | --- | --- | --- | --- | --- | --- | --- | --- | --- | --- | --- | --- | --- | --- | --- | --- | --- | --- | --- | --- | --- | --- | --- | --- | --- | --- | --- | --- | --- | --- | --- | --- | --- | --- | --- | --- | --- | --- | --- |
|  |  |  |  |  |

**Social Sciences Citation Index (SSCI)**

**Science Citation Index Expanded (SCI-EXPANDED)**

**using Web of Science (1970 to present)**

| \| # 40 \| (#39) AND Language=(English) \|  \| [3,242](https://apps.webofknowledge.com/summary.do?product=WOS&doc=1&qid=40&SID=W1eAGdMI9f@o264e9Mj&search_mode=AdvancedSearch) \| \| --- \| --- \| --- \| --- \| \| # 39 \| #38 AND #34 AND #11 \|  \| [3,388](https://apps.webofknowledge.com/summary.do?product=WOS&doc=1&qid=39&SID=W1eAGdMI9f@o264e9Mj&search_mode=CombineSearches) \| \| # 38 \| #37 OR #36 OR #35 \|  \| [5,130,729](https://apps.webofknowledge.com/summary.do?product=WOS&doc=1&qid=38&SID=W1eAGdMI9f@o264e9Mj&search_mode=CombineSearches) \| \| # 37 \| ts=(observ* OR cohort or prospectiv* OR retrospectiv* OR population OR longitud* OR community OR "case* control" OR "cross* section*") \|  \| [5,066,908](https://apps.webofknowledge.com/summary.do?product=WOS&doc=1&qid=37&SID=W1eAGdMI9f@o264e9Mj&search_mode=AdvancedSearch) \| \| # 36 \| ts=general pract* \|  \| [101,275](https://apps.webofknowledge.com/summary.do?product=WOS&doc=1&qid=36&SID=W1eAGdMI9f@o264e9Mj&search_mode=AdvancedSearch) \| \| # 35 \| ts=family pract*,ti,ab \|  \| [2](https://apps.webofknowledge.com/summary.do?product=WOS&doc=1&qid=35&SID=W1eAGdMI9f@o264e9Mj&search_mode=AdvancedSearch) \| \| # 34 \| #33 OR #32 OR #31 OR #30 OR #29 OR #28 OR #27 OR #26 OR #25 OR #24 OR #23 OR #22 OR #21 OR #20 OR #19 OR #18 OR #17 OR #16 OR #15 OR #14 OR #13 OR #12 \|  \| [123,535](https://apps.webofknowledge.com/summary.do?product=WOS&doc=1&qid=34&SID=W1eAGdMI9f@o264e9Mj&search_mode=CombineSearches) \| \| # 33 \| (ts= ("back pain" or "spine pain" or "spinal pain" or "lumbar pain" or "lumbo* pain")) AND Language=(English) \|  \| [30,018](https://apps.webofknowledge.com/summary.do?product=WOS&doc=1&qid=33&SID=W1eAGdMI9f@o264e9Mj&search_mode=AdvancedSearch) \| \| # 32 \| (ts=("foot pain" or "ankle pain" or "elbow pain")) AND Language=(English) \|  \| [1,183](https://apps.webofknowledge.com/summary.do?product=WOS&doc=1&qid=32&SID=W1eAGdMI9f@o264e9Mj&search_mode=AdvancedSearch) \| \| # 31 \| (ts=("knee pain" or "hip pain" or "shoulder pain")) AND Language=(English) \|  \| [7,143](https://apps.webofknowledge.com/summary.do?product=WOS&doc=1&qid=31&SID=W1eAGdMI9f@o264e9Mj&search_mode=AdvancedSearch) \| \| # 30 \| (ts=("neck pain" or "cervical pain")) AND Language=(English) \|  \| [4,716](https://apps.webofknowledge.com/summary.do?product=WOS&doc=1&qid=30&SID=W1eAGdMI9f@o264e9Mj&search_mode=AdvancedSearch) \| \| # 29 \| (ts=("back pain" OR "lumbar pain" OR "lumbo* pain" OR "spine pain" OR "spinal pain")) AND Language=(English) \|  \| [30,018](https://apps.webofknowledge.com/summary.do?product=WOS&doc=1&qid=29&SID=W1eAGdMI9f@o264e9Mj&search_mode=AdvancedSearch) \| \| # 28 \| ts=(musculo* OR muscular near pain) \|  \| [30,199](https://apps.webofknowledge.com/summary.do?product=WOS&doc=1&qid=28&SID=W1eAGdMI9f@o264e9Mj&search_mode=AdvancedSearch) \| \| # 27 \| ts="pain measurement" \|  \| [892](https://apps.webofknowledge.com/summary.do?product=WOS&doc=1&qid=27&SID=W1eAGdMI9f@o264e9Mj&search_mode=AdvancedSearch) \| \| # 26 \| ts="myofascial pain syndromes" \|  \| [134](https://apps.webofknowledge.com/summary.do?product=WOS&doc=1&qid=26&SID=W1eAGdMI9f@o264e9Mj&search_mode=AdvancedSearch) \| \| # 25 \| ts="complex regional pain syndromes" \|  \| [120](https://apps.webofknowledge.com/summary.do?product=WOS&doc=1&qid=25&SID=W1eAGdMI9f@o264e9Mj&search_mode=AdvancedSearch) \| \| # 24 \| ts="non* malignant pain" \|  \| [232](https://apps.webofknowledge.com/summary.do?product=WOS&doc=1&qid=24&SID=W1eAGdMI9f@o264e9Mj&search_mode=AdvancedSearch) \| \| # 23 \| ts="non* cancer pain" \|  \| [277](https://apps.webofknowledge.com/summary.do?product=WOS&doc=1&qid=23&SID=W1eAGdMI9f@o264e9Mj&search_mode=AdvancedSearch) \| \| # 22 \| ts=(comorbid* near pain) \|  \| [1,501](https://apps.webofknowledge.com/summary.do?product=WOS&doc=1&qid=22&SID=W1eAGdMI9f@o264e9Mj&search_mode=AdvancedSearch) \| \| # 21 \| ts=(mult* near pain) \|  \| [12,498](https://apps.webofknowledge.com/summary.do?product=WOS&doc=1&qid=21&SID=W1eAGdMI9f@o264e9Mj&search_mode=AdvancedSearch) \| \| # 20 \| ts="presence of pain" \|  \| [437](https://apps.webofknowledge.com/summary.do?product=WOS&doc=1&qid=20&SID=W1eAGdMI9f@o264e9Mj&search_mode=AdvancedSearch) \| \| # 19 \| ts=(regional near pain) \|  \| [3,406](https://apps.webofknowledge.com/summary.do?product=WOS&doc=1&qid=19&SID=W1eAGdMI9f@o264e9Mj&search_mode=AdvancedSearch) \| \| # 18 \| ts=(radicular near pain) \|  \| [1,558](https://apps.webofknowledge.com/summary.do?product=WOS&doc=1&qid=18&SID=W1eAGdMI9f@o264e9Mj&search_mode=AdvancedSearch) \| \| # 17 \| ts=(joint near pain) \|  \| [11,612](https://apps.webofknowledge.com/summary.do?product=WOS&doc=1&qid=17&SID=W1eAGdMI9f@o264e9Mj&search_mode=AdvancedSearch) \| \| # 16 \| ts=(chronic near pain) \|  \| [36,863](https://apps.webofknowledge.com/summary.do?product=WOS&doc=1&qid=16&SID=W1eAGdMI9f@o264e9Mj&search_mode=AdvancedSearch) \| \| # 15 \| ts=myalgia \|  \| [4,384](https://apps.webofknowledge.com/summary.do?product=WOS&doc=1&qid=15&SID=W1eAGdMI9f@o264e9Mj&search_mode=AdvancedSearch) \| \| # 14 \| ts=fibromyalgia \|  \| [8,943](https://apps.webofknowledge.com/summary.do?product=WOS&doc=1&qid=14&SID=W1eAGdMI9f@o264e9Mj&search_mode=AdvancedSearch) \| \| # 13 \| ts=arthralgia \|  \| [3,282](https://apps.webofknowledge.com/summary.do?product=WOS&doc=1&qid=13&SID=W1eAGdMI9f@o264e9Mj&search_mode=AdvancedSearch) \| \| # 12 \| ts=(widespread NEAR pain) \|  \| [1,613](https://apps.webofknowledge.com/summary.do?product=WOS&doc=1&qid=12&SID=W1eAGdMI9f@o264e9Mj&search_mode=AdvancedSearch) \| \| # 11 \| #10 OR #9 OR #8 OR #7 OR #6 OR #5 OR #4 OR #3 OR #2 OR #1 \|  \| [1,408,621](https://apps.webofknowledge.com/summary.do?product=WOS&doc=1&qid=11&SID=W1eAGdMI9f@o264e9Mj&search_mode=CombineSearches) \| \| # 10 \| ts=survival \|  \| [559,536](https://apps.webofknowledge.com/summary.do?product=WOS&doc=1&qid=10&SID=W1eAGdMI9f@o264e9Mj&search_mode=AdvancedSearch) \| \| # 9 \| ts="sudden death" \|  \| [17,548](https://apps.webofknowledge.com/summary.do?product=WOS&doc=1&qid=9&SID=W1eAGdMI9f@o264e9Mj&search_mode=AdvancedSearch) \| \| # 8 \| ts=died \|  \| [177,790](https://apps.webofknowledge.com/summary.do?product=WOS&doc=1&qid=8&SID=W1eAGdMI9f@o264e9Mj&search_mode=AdvancedSearch) \| \| # 7 \| ts=dead \|  \| [50,560](https://apps.webofknowledge.com/summary.do?product=WOS&doc=1&qid=7&SID=W1eAGdMI9f@o264e9Mj&search_mode=AdvancedSearch) \| \| # 6 \| ts=fatal* \|  \| [70,550](https://apps.webofknowledge.com/summary.do?product=WOS&doc=1&qid=6&SID=W1eAGdMI9f@o264e9Mj&search_mode=AdvancedSearch) \| \| # 5 \| ts="hospital mortality" \|  \| [13,410](https://apps.webofknowledge.com/summary.do?product=WOS&doc=1&qid=5&SID=W1eAGdMI9f@o264e9Mj&search_mode=AdvancedSearch) \| \| # 4 \| ts="fatal outcome" \|  \| [3,360](https://apps.webofknowledge.com/summary.do?product=WOS&doc=1&qid=4&SID=W1eAGdMI9f@o264e9Mj&search_mode=AdvancedSearch) \| \| # 3 \| ts="cause of death" \|  \| [19,405](https://apps.webofknowledge.com/summary.do?product=WOS&doc=1&qid=3&SID=W1eAGdMI9f@o264e9Mj&search_mode=AdvancedSearch) \| \| # 2 \| ts=death \|  \| [458,758](https://apps.webofknowledge.com/summary.do?product=WOS&doc=1&qid=2&SID=W1eAGdMI9f@o264e9Mj&search_mode=AdvancedSearch) \| \| # 1 \| ts=mortality \|  \| [432,078](https://apps.webofknowledge.com/summary.do?product=WOS&doc=1&qid=1&SID=W1eAGdMI9f@o264e9Mj&search_mode=AdvancedSearch) \| |  |  | | |  | |
| --- | --- | --- | --- | --- | --- | --- | --- | --- | --- | --- | --- | --- | --- | --- | --- | --- | --- | --- | --- | --- | --- | --- | --- | --- | --- | --- | --- | --- | --- | --- | --- | --- | --- | --- | --- | --- | --- | --- | --- | --- | --- | --- | --- | --- | --- | --- | --- | --- | --- | --- | --- | --- | --- | --- | --- | --- | --- | --- | --- | --- | --- | --- | --- | --- | --- | --- | --- | --- | --- | --- | --- | --- | --- | --- | --- | --- | --- | --- | --- | --- | --- | --- | --- | --- | --- | --- | --- | --- | --- | --- | --- | --- | --- | --- | --- | --- | --- | --- | --- | --- | --- | --- | --- | --- | --- | --- | --- | --- | --- | --- | --- | --- | --- | --- | --- | --- | --- | --- | --- | --- | --- | --- | --- | --- | --- | --- | --- | --- | --- | --- | --- | --- | --- | --- | --- | --- | --- | --- | --- | --- | --- | --- | --- | --- | --- | --- | --- | --- | --- | --- | --- | --- | --- | --- | --- | --- | --- | --- | --- | --- | --- | --- | --- | --- | --- | --- |
|  |  | |  |  | |  |

**Cochrane library: (Cochrane Database of Systematic Reviews (Cochrane Reviews), Database of Abstracts of Reviews of Effects (Other Reviews)**

| \| #1 \| [(mortality.ti,ab)](http://onlinelibrary.wiley.com/o/cochrane/searchHistory?mode=runquery&qnum=1) \| 9513 \| \| --- \| --- \| --- \| \| #2 \| [mortality/](http://onlinelibrary.wiley.com/o/cochrane/searchHistory?mode=runquery&qnum=2) \| 38650 \| \| #3 \| [cause of death/](http://onlinelibrary.wiley.com/o/cochrane/searchHistory?mode=runquery&qnum=3) \| 8828 \| \| #4 \| [fatal outcome/](http://onlinelibrary.wiley.com/o/cochrane/searchHistory?mode=runquery&qnum=4) \| 2323 \| \| #5 \| [hospital mortality/](http://onlinelibrary.wiley.com/o/cochrane/searchHistory?mode=runquery&qnum=5) \| 16276 \| \| #6 \| [cause of death.ti,ab](http://onlinelibrary.wiley.com/o/cochrane/searchHistory?mode=runquery&qnum=6) \| 3421 \| \| #7 \| [fatal*.ti,ab](http://onlinelibrary.wiley.com/o/cochrane/searchHistory?mode=runquery&qnum=7) \| 9513 \| \| #8 \| [death.ti,ab](http://onlinelibrary.wiley.com/o/cochrane/searchHistory?mode=runquery&qnum=8) \| 9513 \| \| #9 \| [died.ti,ab](http://onlinelibrary.wiley.com/o/cochrane/searchHistory?mode=runquery&qnum=9) \| 9513 \| \| #10 \| [death/](http://onlinelibrary.wiley.com/o/cochrane/searchHistory?mode=runquery&qnum=10) \| 24789 \| \| #11 \| [death,sudden/](http://onlinelibrary.wiley.com/o/cochrane/searchHistory?mode=runquery&qnum=11) \| 25554 \| \| #12 \| [dead.ti,ab](http://onlinelibrary.wiley.com/o/cochrane/searchHistory?mode=runquery&qnum=12) \| 9513 \| \| #13 \| [survival/](http://onlinelibrary.wiley.com/o/cochrane/searchHistory?mode=runquery&qnum=13) \| 36976 \| \| #14 \| [survival.ti,ab](http://onlinelibrary.wiley.com/o/cochrane/searchHistory?mode=runquery&qnum=14) \| 9513 \| \| #15 \| [widespread adj3 pain.ti,ab](http://onlinelibrary.wiley.com/o/cochrane/searchHistory?mode=runquery&qnum=15) \| 141 \| \| #16 \| [arthralgia.ti,ab](http://onlinelibrary.wiley.com/o/cochrane/searchHistory?mode=runquery&qnum=16) \| 9513 \| \| #17 \| [fibromyalgia.ti,ab](http://onlinelibrary.wiley.com/o/cochrane/searchHistory?mode=runquery&qnum=17) \| 9513 \| \| #18 \| [myalgia.ti,ab](http://onlinelibrary.wiley.com/o/cochrane/searchHistory?mode=runquery&qnum=18) \| 9513 \| \| #19 \| [chronic adj3 pain.ti,ab](http://onlinelibrary.wiley.com/o/cochrane/searchHistory?mode=runquery&qnum=19) \| 564 \| \| #20 \| [joint adj pain.ti,ab](http://onlinelibrary.wiley.com/o/cochrane/searchHistory?mode=runquery&qnum=20) \| 346 \| \| #21 \| [radicular adj pain.ti,ab](http://onlinelibrary.wiley.com/o/cochrane/searchHistory?mode=runquery&qnum=21) \| 31 \| \| #22 \| [regional adj3 pain.ti,ab](http://onlinelibrary.wiley.com/o/cochrane/searchHistory?mode=runquery&qnum=22) \| 218 \| \| #23 \| [presence of pain.ti,ab](http://onlinelibrary.wiley.com/o/cochrane/searchHistory?mode=runquery&qnum=23) \| 2168 \| \| #24 \| [mult* adj3 pain.ti,ab](http://onlinelibrary.wiley.com/o/cochrane/searchHistory?mode=runquery&qnum=24) \| 884 \| \| #25 \| [musculoskeletal pain/](http://onlinelibrary.wiley.com/o/cochrane/searchHistory?mode=runquery&qnum=25) \| 1887 \| \| #26 \| [chronic pain/](http://onlinelibrary.wiley.com/o/cochrane/searchHistory?mode=runquery&qnum=26) \| 8610 \| \| #27 \| [exp joint pain/](http://onlinelibrary.wiley.com/o/cochrane/searchHistory?mode=runquery&qnum=27) \| 662 \| \| #28 \| [fibromyalgia/](http://onlinelibrary.wiley.com/o/cochrane/searchHistory?mode=runquery&qnum=28) \| 930 \| \| #29 \| [pain measurement/](http://onlinelibrary.wiley.com/o/cochrane/searchHistory?mode=runquery&qnum=29) \| 17607 \| \| #30 \| [non* cancer pain.ti,ab](http://onlinelibrary.wiley.com/o/cochrane/searchHistory?mode=runquery&qnum=30) \| 1659 \| \| #31 \| [non* malignant pain.ti,ab](http://onlinelibrary.wiley.com/o/cochrane/searchHistory?mode=runquery&qnum=31) \| 524 \| \| #32 \| [arthralgia/](http://onlinelibrary.wiley.com/o/cochrane/searchHistory?mode=runquery&qnum=32) \| 997 \| \| #33 \| [complex regional pain syndromes/](http://onlinelibrary.wiley.com/o/cochrane/searchHistory?mode=runquery&qnum=33) \| 290 \| \| #34 \| [myofascial pain syndromes/](http://onlinelibrary.wiley.com/o/cochrane/searchHistory?mode=runquery&qnum=34) \| 379 \| \| #35 \| [neck or cervical adj3 pain.ti,ab](http://onlinelibrary.wiley.com/o/cochrane/searchHistory?mode=runquery&qnum=35) \| 10404 \| \| #36 \| [knee* or hip or hips or shoulder* adj3 pain.ti,ab](http://onlinelibrary.wiley.com/o/cochrane/searchHistory?mode=runquery&qnum=36) \| 15826 \| \| #37 \| [foot or feet or ankle* or elbow* adj3 pain.ti,ab](http://onlinelibrary.wiley.com/o/cochrane/searchHistory?mode=runquery&qnum=37) \| 7150 \| \| #38 \| [back or lumbar or lumbo* or spine or spinal adj3 pain.ti,ab](http://onlinelibrary.wiley.com/o/cochrane/searchHistory?mode=runquery&qnum=38) \| 16659 \| \| #39 \| [musculo* or muscular adj3 pain.ti,ab](http://onlinelibrary.wiley.com/o/cochrane/searchHistory?mode=runquery&qnum=39) \| 3443 \| \| #40 \| [longitudinal studies/](http://onlinelibrary.wiley.com/o/cochrane/searchHistory?mode=runquery&qnum=40) \| 7733 \| \| #41 \| [cohort studies/](http://onlinelibrary.wiley.com/o/cochrane/searchHistory?mode=runquery&qnum=41) \| 18545 \| \| #42 \| [prospective studies/](http://onlinelibrary.wiley.com/o/cochrane/searchHistory?mode=runquery&qnum=42) \| 96203 \| \| #43 \| [family practice/ or general practice/](http://onlinelibrary.wiley.com/o/cochrane/searchHistory?mode=runquery&qnum=43) \| 18426 \| \| #44 \| [retrospective studies/](http://onlinelibrary.wiley.com/o/cochrane/searchHistory?mode=runquery&qnum=44) \| 12135 \| \| #45 \| [Case-Control Studies/ or Epidemiologic Methods/](http://onlinelibrary.wiley.com/o/cochrane/searchHistory?mode=runquery&qnum=45) \| 8036 \| \| #46 \| [Cross-Sectional Studies/](http://onlinelibrary.wiley.com/o/cochrane/searchHistory?mode=runquery&qnum=46) \| 5307 \| \| #47 \| [family pract*.ti,ab](http://onlinelibrary.wiley.com/o/cochrane/searchHistory?mode=runquery&qnum=47) \| 9513 \| \| #48 \| [general pract*.ti,ab](http://onlinelibrary.wiley.com/o/cochrane/searchHistory?mode=runquery&qnum=48) \| 9513 \| \| #49 \| [observ* or cohort or prospectiv* or retrospectiv* or population or longitud* or community or case* control or cross* section*.ti,ab.](http://onlinelibrary.wiley.com/o/cochrane/searchHistory?mode=runquery&qnum=49) \| 267548 \| \| #50 \| [(#1 OR #2 OR #3 OR #4 OR #5 OR #6 OR #7 OR #8 OR #9 OR #10 OR #11 OR #12 OR #13 OR #14)](http://onlinelibrary.wiley.com/o/cochrane/searchHistory?mode=runquery&qnum=50) \| 74562 \| \| #51 \| [(#15 OR #16 OR #17 OR #18 OR #19 OR #20 OR #21 OR #22 OR #23 OR #24 OR #25 OR #26 OR #27 OR #28 OR #29 OR #30 OR #31 OR #32 OR #33 OR #34 OR #35 OR #36 OR #37 OR #38 OR #39)](http://onlinelibrary.wiley.com/o/cochrane/searchHistory?mode=runquery&qnum=51) \| 68424 \| \| #52 \| [(#40 OR #41 OR #42 OR #43 OR #44 OR #45 OR #46 OR #47 OR #48 OR #49)](http://onlinelibrary.wiley.com/o/cochrane/searchHistory?mode=runquery&qnum=52) \| 275080 \| \| #53 \| [(#50 AND #51 AND #52)](http://onlinelibrary.wiley.com/o/cochrane/searchHistory?mode=runquery&qnum=53) \| 13097 \| \| #54 \| [(#53)](http://onlinelibrary.wiley.com/o/cochrane/searchHistory?mode=runquery&qnum=54) \| **5104** \| |  |  |
| --- | --- | --- | --- | --- | --- | --- | --- | --- | --- | --- | --- | --- | --- | --- | --- | --- | --- | --- | --- | --- | --- | --- | --- | --- | --- | --- | --- | --- | --- | --- | --- | --- | --- | --- | --- | --- | --- | --- | --- | --- | --- | --- | --- | --- | --- | --- | --- | --- | --- | --- | --- | --- | --- | --- | --- | --- | --- | --- | --- | --- | --- | --- | --- | --- | --- | --- | --- | --- | --- | --- | --- | --- | --- | --- | --- | --- | --- | --- | --- | --- | --- | --- | --- | --- | --- | --- | --- | --- | --- | --- | --- | --- | --- | --- | --- | --- | --- | --- | --- | --- | --- | --- | --- | --- | --- | --- | --- | --- | --- | --- | --- | --- | --- | --- | --- | --- | --- | --- | --- | --- | --- | --- | --- | --- | --- | --- | --- | --- | --- | --- | --- | --- | --- | --- | --- | --- | --- | --- | --- | --- | --- | --- | --- | --- | --- | --- | --- | --- | --- | --- | --- | --- | --- | --- | --- | --- | --- | --- | --- | --- | --- | --- | --- | --- |

**Ageline using EBSCO (1978 to present)**

| S33 | S9 and S27 and S32 | | | 77 |
| --- | --- | --- | --- | --- |
| S32 | S28 or S29 or S30 or S31 | | | 51780 |
| S31 | observ* or cohort or prospectiv* or retrospectiv* or population or longitud* or community or case* control or cross* section* | | | 51142 |
| S30 | family pract* | | | 616 |
| S29 | general pract* | | | 778 |
| S28 | (DE "Longitudinal Studies") OR (DE "Cohorts") | | | 6281 |
| S27 | S10 or S11 or S12 or S13 or S14 or S15 or S16 or S17 or S18 or S19 or S20 or S21 or S22 or S23 or S24 or S25 or S26 | | | 1294 |
| S26 | (BACK or LUMBAR or LUMBO* or SPINE or SPINAL) and PAIN | | | 203 |
| S25 | (FOOT or FEET or ANKLE* or ELBOW*) and PAIN | | | 63 |
| S24 | (KNEE* or HIP or HIPS or SHOULDER*) and PAIN | | | 173 |
| S23 | (NECK or CERVICAL) and PAIN | | | 40 |
| S22 | Myofascial pain syndrome | | | 1 |
| S21 | (musculo* or muscular) and pain | | | 97 |
| S20 | comorbid* and pain | | | 119 |
| S19 | mult* and pain | | | 506 |
| S18 | "presence of pain" | | | 18 |
| S17 | regional and pain | | | 22 |
| S16 | joint and pain | | | 189 |
| S15 | chronic and pain | | | 541 |
| S14 | Myalgia | | | 2 |
| S13 | Fibromyalgia | | | 20 |
| S12 | Arthralgia | | | 1 |
| S11 | widespread and pain | | | 20 |
| S10 | DE "Chronic Pain" | | | 209 |
| S9 | S1 or S2 or S3 or S4 or S5 or S6 or S7 or S8 | | | 11051 |
| S8 | Survival | | | 1486 |
| S7 | Died | | | 1455 |
| S6 | Dead | | | 211 |
| S5 | Death | | | 8341 |
| S4 | fatal* | | | 339 |
| S3 | "cause of death" | | | 229 |
| S2 | DE "Death" OR DE "Death Causes" OR DE "Death Rates" | | | 3519 |
| S1 | Mortality | | | 3532 |
|  |  | | |  |
| Database | No of items | Duplicates per database | Total Duplicates | Total items |
| Ageline | 77 | 0 | 0 | 77 |
| AMED | 42 | 0 | 0 | 119 |
| CINAHL | 3232 | 33 | 33 | 3318 |
| Cochrane | 4775 | 48 | 81 | 8045 |
| DARE | 329 | 8 | 89 | 8366 |
| EMBASE | 4651 | 394 | 483 | 12623 |
| Medline | 1935 | 1478 | 1961 | 13080 |
| PsychInfo | 100 | 61 | 2022 | 13119 |
| Web of Science | 3242 | 1304 | 3326 | 15057 |
| Total | 18383 | 3326 | 3326 | 15057 |
